# NHEJ pathway is involved in post-integrational DNA repair due to Ku70 binding to HIV-1 integrase

**DOI:** 10.1186/s12977-019-0492-z

**Published:** 2019-11-06

**Authors:** Ekaterina Knyazhanskaya, Andrey Anisenko, Olga Shadrina, Anastasia Kalinina, Timofei Zatsepin, Arthur Zalevsky, Dmitriy Mazurov, Marina Gottikh

**Affiliations:** 1 Chemistry Department, Lomonosov Moscow State University, Moscow, 199234 Russia; 2 Belozersky Institute of Physico-Chemical Biology, Lomonosov Moscow State University, Moscow, 119234 Russia; 3 Faculty of Bioengineering and Bioinformatics, Lomonosov Moscow State University, Moscow, 119234 Russia; 4 Federal State Budgetary Institution « N.N. Blokhin National Medical Research Center of Oncology » of the Ministry of Health of the Russian Federation, Moscow, 115478 Russia; 5 Skolkovo Institute of Science and Technology, Skolkovo, 121205 Russia; 6 Center for Precision Genome Editing and Genetic Technologies for Biomedicine, Institute of Gene Biology, RAS, Moscow, 119334 Russia; 7 NRC Institute of Immunology FMBA of Russia, Moscow, 115478 Russia; 8 Present Address: Department of Biochemistry and Molecular Biology, Sealy Center for Structural Biology and Molecular Biophysics, University of Texas Medical Branch, Galveston, TX 77555 USA

**Keywords:** HIV-1, Integrase, NHEJ, Ku70, DNA-PK, Post integrational gap repair

## Abstract

**Background:**

HIV-1 integration results in genomic DNA gaps that are repaired by cellular DNA repair pathways. This step of the lentiviral life cycle remains poorly understood despite its crucial importance for successful replication. We and others reported that Ku70 protein of the non-homologous end joining pathway (NHEJ) directly binds HIV-1 integrase (IN). Here, we studied the importance of this interaction for post-integrational gap repair and the recruitment of NHEJ factors in this process.

**Results:**

We engineered HIV-based pseudovirus with mutant IN defective in Ku70 binding and generated heterozygous Ku70, Ku80 and DNA-PKcs human knockout (KO) cells using CRISPR/Cas9. KO of either of these proteins or inhibition of DNA-PKcs catalytic activity substantially decreased the infectivity of HIV-1 with native IN but not with the mutant one. We used a recently developed qPCR assay for the measurement of gap repair efficiency to show that HIV-1 with mutant IN was defective in DNA post-integrational repair, whereas the wild type virus displayed such a defect only when NHEJ system was disrupted in any way. This effect was present in CRISPR/Cas9 modified 293T cells, in Jurkat and CEM lymphoid lines and in primary human PBMCs.

**Conclusions:**

Our data provide evidence that IN recruits DNA-PK to the site of HIV-1 post-integrational repair due to Ku70 binding—a novel finding that explains the involvement of DNA-PK despite the absence of free double stranded DNA breaks. In addition, our data clearly indicate the importance of interactions between HIV-1 IN and Ku70 in HIV-1 replication at the post-integrational repair step.

## Background

**Fig. 1:**
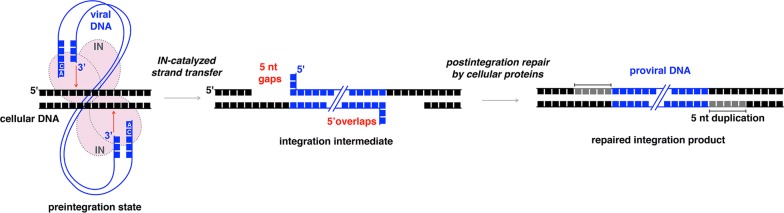
A schematic illustration of retroviral integration. Structure of the integration intermediate and post-integrational DNA repair are present

Integration of viral DNA into a host genome is an important step of HIV-1 replication cycle. It is performed by viral enzyme integrase (IN) that being in a multimeric form [1–3], binds viral DNA and catalyzes the cleavage of dinucleotides from both its 3′-ends. The 3′-processed viral DNA in complex with IN and a number of viral and cellular proteins is then transported into nucleus, where IN catalyzes the second step of integration by inserting each of the processed viral DNA 3′-ends into one strand of cellular DNA [4, 5]. This insertion results in the formation of 5 nucleotide gaps [5–7]. Consequently, 3′-ends of viral DNA are covalently linked to the cellular DNA, whereas the 5′-ends form an overhang due to an unpaired dinucleotide (Fig. 1). To complete integration, restore genome integrity and enable virus replication, this integration intermediate has to be repaired [8]. It has been generally implied, that this step is performed by the cellular DNA repair machinery [5, 8–10]. However, the precise mechanism of the integration intermediate repair is not yet clear.

A screen of knockout libraries showed that numerous DNA repair pathways participate in HIV-1 replication, i.e. base and nucleoside excision, homologous recombination (HR) and non-homologous end joining (NHEJ) [11–14]. The involvement of the base excision repair (BER) pathway was in part confirmed by biochemical and genetic studies [15–17]. RAD51 protein, which is an important player in HR, was found to affect HIV-1 integration [18, 19]. The involvement of the NHEJ pathway in HIV-1 replication has undergone particularly extensive studies providing numerous data showing that depletion of proteins involved in this pathway decreases HIV-1 infectivity [9, 20–25]. Nonetheless, the precise mode of NHEJ pathway participation in the HIV-1 replication remains obscure. In particular, there is no direct evidence showing that it is exactly the post-integrational gap repair step that is performed by NHEJ components as well as by HR or BER repair systems but rather this conclusion is usually extrapolated from the fact that all three are DNA-repair systems.

Classical NHEJ is initiated by the binding of Ku heterodimer to free dsDNA ends emerging at the site of a double strand break (DSB) [8, 26, 27]. This complex recruits the catalytic subunit of DNA-dependent protein kinase (DNA-PKcs), which triggers a cascade of phosphorylation events [28] and the subsequent DSB repair performed by such downstream factors as Artemis and DNA Ligase IV in complex with XRCC4 and XLF co-factors [29–31]. Ku heterodimer consists of Ku70 and Ku80 subunits. The positive role of Ku80 for lentiviral replication has been shown in several works [22–24] and attributed either to transcription of integrated provirus [23] or integration of HIV-1 DNA [22]. Ku70 has been reported to form a stable complex with HIV-1 IN [25, 32, 33] and to increase its proteolytic stability thus stimulating HIV-1 replication [25], but screening experiments with knockdown or knockout of a set of human proteins have not confirmed a negative effect of Ku70 depletion on HIV-1 infectivity [14, 34]. The importance of an active DNA-PKcs for an efficient lentiviral infection has been first shown as early as 1999 [20] and in several later studies [21, 35]. A knockout of DNA-PKcs or an inhibition of its catalytic activity not only reduces viral replication, but also affects survival of infected cells [21, 36]. This cytotoxic effect can be rescued either by restoring the DNA-PKcs expression [21] or by using a virus with a catalytically inactive IN [20, 21, 36], the latter pointing to DNA-PK involvement in lentiviral integration process. However, the role of DNA-PKcs in the HIV-1 DNA integration is denied in [37, 38].

Therefore, there are two major questions regarding the involvement of NHEJ in HIV-1 post-integrational gap repair: (1) Is the repair of the integration intermediate a specific replication step that is affected by a decrease in the components of DNA-PK? (2) Considering that free dsDNA ends cannot be produced during HIV-1 integration (Fig. 1), what is the mechanism of Ku recruitment to the integration site?

The first question has not been answered so far because in previous works the exact amount of repaired integration intermediate was not analyzed due to the lack of an appropriate protocol. We have developed a modified version of Alu-specific qPCR assay for the quantification of HIV-1 post-integrational gap repair efficiency [39]. Here, we used the modified qPCR to quantify the repair levels in a single-cycle transduction assay and unambiguously demonstrated for the first time that the knockout of major players of NHEJ system, e.g. Ku70, Ku80 or DNA-PKcs, substantially reduced the level of post-integrational DNA gap repair, which was in good correlation with a decrease in HIV-1 infectivity. Moreover, an efficient DNA gap repair was found to require IN binding to Ku70 since mutant HIV-1 with IN bearing E212A/L213A substitutions, which are critical for this binding [33], was defective exactly at the gap repair step. The transduction efficiency of the mutant virus was reduced in wild type cells but was insensitive to the depletion of Ku70, Ku80 or DNA-PKcs. Thus, according to our data Ku is recruited to the integration site through an interaction with IN. This finding provided us with an answer for the second question. Altogether, these data allowed us to propose a model where the direct interaction of Ku70 with HIV-1 IN provides the recruitment of other components of DNA-PK complex to the integration site, which in turn triggers post-integrational DNA gap repair by the NHEJ pathway and enables efficient viral replication.

## Results

### The stabilization of HIV-1 IN by Ku70 does not require a direct interaction between two proteins

**Fig. 2:**
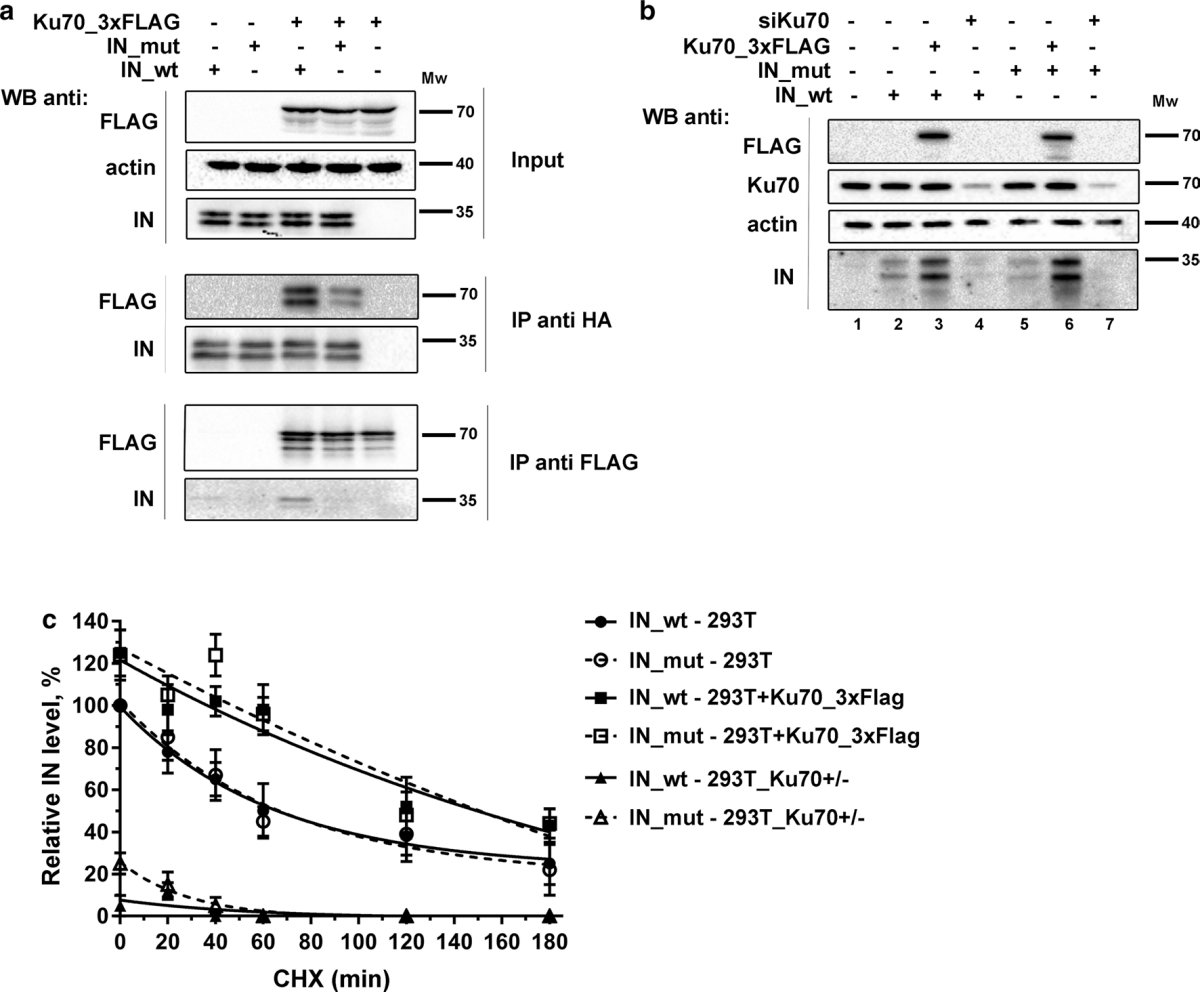
The effect of Ku70 on HIV-1 integrase stability in 293T cells. **a** Immunoprecipitation of HIV-1 IN_HA on anti-HA antibody conjugated beads (middle panel) or of Ku70_3×FLAG on anti-FLAG antibody conjugated beads (lower panel). IN_wt or IN_mut were expressed in 293T cells with Ku70_3×FLAG or with an empty vector, then cells were lysed, 10% of lysates were saved for input analysis (upper panel), the rest was divided in two and subjected to immunoprecipitation for 4 h at 4 °C, then the beads were washed 4 times with incubation buffer and proteins were eluted by 0.1 M glycine, pH 2.5, and the eluates together with input samples were analyzed by Western blot. Representative images of at least three independent experiments are shown. **b** Western blot analysis of a superexpression of IN_wt (lanes 2–4) or IN_mut (lanes 5–7) either in the presence of superexpressed Ku70_3×FLAG (lanes 3 and 6) or siRNA for Ku70 (lanes 4 and 7) or an empty vector (lanes 2 and 5). Representative image of at least three independent experiments is shown. **c** Plot showing IN stability in 293T cells upon cycloheximide treatment. IN relative amount was determined as band intensity normalized to tubulin. Mean values ± SD (n = 3) are shown. The weight marker mobility levels are presented to the right of the WB panels

It has been shown that a decrease in the intracellular concentration of Ku reduces HIV-1 replication [22, 23, 25, 33]. This effect can be explained either by an IN protection from proteasomal degradation by Ku70 [25], a Ku80-mediated stimulation of HIV-1 transcription [23] or a participation of Ku in the post-integrational gap repair. The Ku70 protective effect may arise from a direct shielding of HIV-1 IN in its complex with Ku70 [25]. Earlier, we have found that HIV-1 IN bearing substitutions E212A/L213A (IN_mut) shows a weaker binding with Ku70 [33]: an effect observed both on recombinant proteins expressed in bacteria and on proteins that are overexpressed in human cells (Fig. 2a). Of note, in our hands IN also bound to Ku80 in cell lysates, but the binding occurred to similar extent both for wild type IN (IN_wt) and for IN_mut (Additional file 1: Figure S1A). To verify if IN binding to Ku70 indeed protects it from proteasomal degradation, we expressed IN_wt or IN_mut tagged with HA epitope in 293T cells, analyzed protein expression and found no differences in the expression levels of IN with or without mutation (Fig. 2b, c). When Ku70 was transiently overexpressed in cells, the amounts of both IN_wt and IN_mut were elevated. Conversely, siRNA mediated knockdown of Ku70 led to a significant decrease in the levels of both variants of IN (Fig. 2b). Overexpression of Ku80 in 293T cell culture did not noticeably affect the levels of IN—both wt and mut (Additional file 1: Figure S1B), whereas a slight reduction in the IN levels observed under Ku80 knockdown likely resulted from the corresponding decrease of the Ku70 intracellular amount—a well-known effect that has been described previously [23, 40].

To obtain more convincing data on protein stability, we measured the dynamics of IN degradation in the presence of translation inhibitor cycloheximide (Fig. 2c). Under these conditions, IN_wt degraded quickly with a half-life of 65 ± 10 min that is consistent with previous report [41]. The half-life of the IN_mut was similar to that determined for the IN_wt. Ku70 overexpression increased the half-life of both IN_wt and IN_mut to a similar extent (120 ± 15 min, Fig. 2c, Additional file 1: Figure S1C), whereas monoallelic gene knockout of Ku70 (see below) resulted in a sixfold decrease in their expression. It should be noted, that it is the IN protein stability that is affected by the changes in Ku70 intracellular concentration since the IN mRNA levels remained constant in cells with different amount of Ku70 (Additional file 1: Figure S1D). Therefore, HIV-1 IN is indeed stabilized by Ku70 but this effect does not depend on direct binding between these two proteins.

### HIV-1 infectivity depends on the interaction between IN and Ku70

**Fig. 3:**
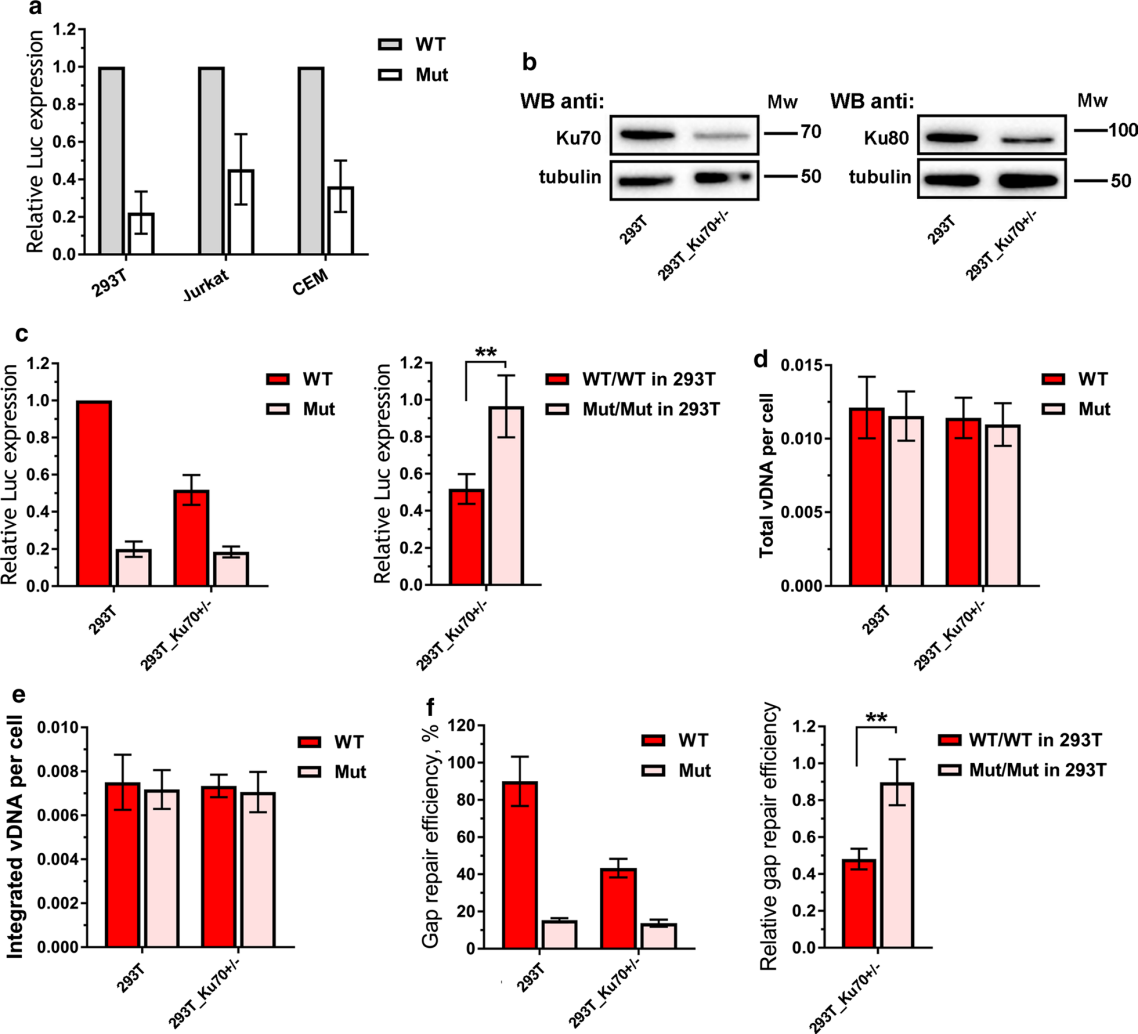
The influence of Ku70 intracellular level on the early replicative stages of HIV-1. **a** Analysis of relative luciferase expression in different cell lines. 293T cells were transduced with 0.01 MOI of either HIV_wt or HIV_mut and the luciferase expression was assayed 24 h later. Jurkat and CEM cell lines were spinoculated with 0.1 MOI of either HIV_wt or HIV_mut in the presence of 7 µg/mL polybrene and the luciferase expression was assayed 24 h later. The results were normalized to the luciferase expression from HIV_wt in 293T cells. Mean values ± SD of at least three independent experiments are presented. **b** Western blots of wild type 293T cells and 293T-Ku70^+/−^ cells probed for Ku70, Ku80 and tubulin as a loading control. **c** Analysis of relative luciferase expression in 293T or 293T-Ku70^+/−^ cells transduced by either HIV_wt or HIV_mut. Left plot: mean values normalized to luciferase expression in 293T cells transduced by HIV_wt ± SD (n = 7); right plot: mean values in 293T-Ku70^+/−^ cells normalized to the data in parental 293T cells for HIV_wt and HIV_mut separately, ± SD (n = 7). **d**, **e** Total viral DNA (**d**) and integrated viral DNA (**e**) in 293T or 293T-Ku70^+/−^ cells transduced by either HIV_wt or HIV_mut measured by a standard qPCR analysis [45]; data was normalized to the data for CD3 gene to achieve per cell normalization. **f** Gap repair efficiency in 293T or 293T-Ku70^+/−^ cells transduced by either HIV_wt or HIV_mut measured by a modified qPCR [39]. Data is shown as percentage of repaired proviral DNA relative to integrated proviral DNA (left plot) or as a repair efficiency of both pseudoviruses in 293T-Ku70^+/−^ cells normalized to the repair efficiency in 293T cells (right plot). **d**–**f** Mean values ± SD of three independent experiments are presented. Significance was determined by two-tailed Student’s *t*-test, ** = p < 0.01, *** = p < 0.001. The weight marker mobility levels are presented to the right of the WB panels

To gain some insight into the physiological significance of the interaction between HIV-1 IN and Ku70, we used a single-round luciferase expressing HIV-based pseudoviruses containing native IN (HIV_wt) or IN with E212A/L213A substitutions defective in Ku70 binding (HIV_mut) [33]. These pseudoviruses were used to infect cells at low MOI (< 0.1) to prevent viral cytotoxicity and/or a potential hyper activation of NHEJ repair machinery [20, 37]. The levels of HIV-1 single cycle infection were quantified by measuring luciferase activity. As shown in Fig. 3a, the infectivity levels for HIV_mut in 293T, Jurkat and CEM cells were three- to sevenfold lower than for HIV_wt suggesting that the HIV-1 life cycle may depend on the interaction between Ku70 and IN. Importantly, although in vitro catalytic activity of IN bearing E212A and L213A mutations was found to be slightly decreased [33], both pseudoviruses, HIV_wt and HIV_mut, provided the same level of DNA integration determined by qPCR assay (see below, Fig. 3e). Thus, the reduced transduction efficiency for HIV_mut could not be explained by the influence of the mutations on the IN activity.

Earlier, we have mapped the N-terminal domain of Ku70 (a.a. 1–250) as the one forming the main IN-binding site [33]. To test whether a truncated Ku70 protein representing the N-terminal domain (Ku70_1–250) would have a dominant negative effect on HIV-1 replication, we transiently expressed Ku70_wt, Ku70_1–250 or Ku70_251–609 and Ku80 alone or in combination with Ku70_wt in 293T cells (Additional file 1: Figure S2) and measured luciferase expression upon pseudoviral transduction. We found that the replication of HIV_wt or HIV_mut was not significantly affected by any of these proteins except for the Ku70_1–250 protein (Additional file 1: Figure S2). The expression of Ku70_1–250 reduced the transduction efficiency of HIV_wt but had no effect on HIV_mut. The inhibition was mild, but still detectable, especially if considering that Ku70_1–250 showed a decreased intracellular stability in comparison to Ku70_wt or Ku70_251–609 (Additional file 1: Figure S2). These data indicate that the Ku70 N-terminal domain interferes with HIV_wt replication presumably by competing with endogenous Ku70 for binding with IN_wt and not with IN_mut defective in Ku70 binding.

In addition to overexpression, we evaluated HIV-1 infectivity levels in cells with Ku70 depletion. For this purpose, using CRISPR/Cas9 technique and SORTS (Surface Oligopeptide knock-in for Rapid Target Selection) method for isolation of gene-edited cells [42] we generated 293T-Ku70^+/−^ cells with monoallelic gene knockout (mKO), in which endogenous level of Ku70 was reduced in comparison to that in parental cells—both on protein and RNA level (Fig. 3b, Additional file 1: Figure S3A). We tried to generate a homozygous clone by cloning the heterogenous CRISPR-treated population after tag sorting, but none of the clones tested were negative for Ku70 expression (Additional file 1: Figure S3B). In further experiments, we used the 293T-Ku70^+/−^ clone 1 that showed the lowest Ku70 expression. As demonstrated in Fig. 3c, the level of HIV_wt transduction efficiency in 293T-Ku70^+/−^ cells was decreased twice relative to that measured in parental cells, which is in consistency with results reported by other groups and us [23, 25, 33]. Unlike it was for HIV_wt, the low infectivity levels detected in 293T cells for HIV_mut were not influenced by Ku70 mKO. The overexpression of Ku70 from plasmid vector in 293T-Ku70^+/−^ cells resulted in the increase of luciferase expression for HIV_wt, but not for HIV_mut (Additional file 1: Fig. S2C). Thus, together with overexpression experiments, these results indicate that Ku70 molecular interaction with IN is important for HIV-1 infectivity.

### The E212A/L213A substitutions in HIV-1 integrase have a negative effect on post-integrational gap repair

To precisely establish the replication step that is influenced by Ku70 mKO and E212A/L213A substitutions in IN, we measured the amount of total reverse transcribed DNA and integrated DNA in cells 24 h after transduction and found both values to be unaffected by the drop in Ku70 level (Fig. 3d, e). Of note, it was true for both HIV_wt and HIV_mut. In our vectors the transgene expression from proviral DNA is driven by CMV promoter that has been previously shown to be independent from Ku level [23]. We confirmed it in our hands using a plasmid vector expressing firefly luciferase under the control of CMV promoter that demonstrated the same level of luciferase expression in parental 293T and 293T-Ku70^+/−^ cells (Additional file 1: Figure S4). Thus, a decrease in HIV-1 infectivity measured by one cycle replication assay (Fig. 3a) cannot be explained by an effect that Ku70 could have on reverse transcription, integration or regulation of transcription from a CMV promoter.

Since Ku70 is an important component of the NHEJ DNA repair pathway, it has been generally assumed that it can be involved in the repair of viral integration intermediates. However, due to a lack of an appropriate quantitative protocol, the precise level of integrated repaired proviral DNA (I-R) has never been measured and reported. Recently, we developed a modified version of Alu-specific PCR that allowed us to perform an accurate quantification of I-R product ([39] and Additional file 1: Figure S5). Briefly, the protocol exploits the fact that the integrated unrepaired proviral DNA (I-U) contains gaps in both strands. We introduced a linear preamplification step in the protocol using U3 annealing primer that amplifies only the I-R proviruses. Then a standard Alu-specific PCR is performed using either the pre-amplified sample or the original one. The difference between these two probes can be simply converted to the fraction of the I-U proviruses among the total integrated proviral DNA.

Using this approach, we compared the post-integrational gap repair efficiency in 293T cells infected with HIV_wt or HIV_mut. As demonstrated in Fig. 3f (left two bars), the level of post-integrational gap repair 24 h post transduction was approximately 5 times lower for HIV_mut than for HIV_wt. This result correlates well with the observed decrease in luciferase expression caused by mutation in IN (Fig. 3a, c). To be sure that the post-integrational gap repair is the only stage influenced by E212A/L213A substitutions in IN, we additionally measured the amount of total reverse transcribed, integrated, and repaired DNA at different time points (Additional file 1: Fig. S6). The data obtained clearly indicate in favor of the substitutions’ effect only on the repair stage. The 293T cell division takes place every 24 h. The single-strand DNA gaps should be repaired after completion of the next S-phase or otherwise the cell would die via activation of various signaling pathways [43]. Taking it into account, we measured the levels of total and integrated viral DNA as well as luciferase expression and gap repair efficiency 72 h post transduction (Additional file 1: Fig. S6). We found no difference in gap repair efficiency for HIV_wt and HIV_mut at this time point (Additional file 1: Fig. S6D), whereas the level of luciferase expression from HIV_mut remains considerably lower than from HIV_wt (Additional file 1: Fig. S6A).

Interestingly, Ku70 mKO resulted in twofold decrease in I-R DNA formation after infection with HIV_wt (Fig. 3f, compare red bars). In contrast, the low level of I-R DNA produced by HIV_mut was unaffected by Ku70 mKO (Fig. 3f, pink bars). When we normalized the data obtained in 293T-Ku70^+/−^ cells to that measured in parental 293T cells, the relative efficiency of I-R product formation was higher for HIV-1 with IN_mut than for HIV_wt (Fig. 3f, right plot). Thus, a decrease in HIV-1 transduction efficiency that resulted from either IN E212A/L213A mutation or Ku70 mKO correlated well with a decrease in HIV-1 I-R proviral DNA formation.

According to a classification proposed earlier, all IN mutants can be divided into two classes: class-1 IN mutants are selectively defective for integration, whereas viruses with class-2 IN mutants are characterized by pleiotropic defects, including virion assembly and/or reverse transcription [44]. E212A/L213A substitutions in IN resulted in viral defects in neither reverse transcription nor integration (Fig. 3d, e) but led to a reduced post-integrational DNA repair (Fig. 3f). Since this is a rather unusual case, we decided to verify the approaches that we used for measuring viral replication defects. For this purpose, we prepared control pseudoviruses containing well-characterized class-1 IN mutant E152A [38] (HIV_E152A) and two class-2 IN mutants, H16C [45] and F185A [46] (HIV_H16C and HIV_F185A, respectively). All the control pseudoviruses demonstrated reduced luciferase expression (Additional file 1: Figure S7A), although we used a higher MOI (> 1) for reliable determination of the luciferase expression level in the case of the control pseudoviruses. In the case of HIV_E152A bearing catalytically inactive IN the amount of total reverse transcribed DNA in cells 24 h after transduction was unaffected, whereas the level of integrated DNA was significantly reduced (Additional file 1: Figure S7B and C). For HIV_H16C and HIV_F185A we detected a dramatic decrease in the amount of total reverse transcribed DNA that led to an almost undetectable level of integrated DNA (Additional file 1: Figure S7B and C). Due to extremely low levels of integrated DNA, we failed to determine the efficiency of I-R DNA formation after infection for all of the control pseudoviruses. Altogether these data showed that HIV-1 IN bearing E212A/L213A substitutions indeed differed from the well-characterized class-1 and class-2 IN mutants.

In summary, using a new qPCR-based approach allowing the quantification of HIV-1 I-R proviral DNA, we established that it is the post-integrational gap repair step in HIV-1 life cycle that is affected by interaction of viral IN with Ku70 or Ku70 depletion. These data point to the NHEJ pathway involvement in HIV-1 post-integrational repair process.

### Ku70, Ku80 and DNA-PKcs are all important for HIV-1 infectivity and post-integrational DNA gap repair

**Fig. 4:**
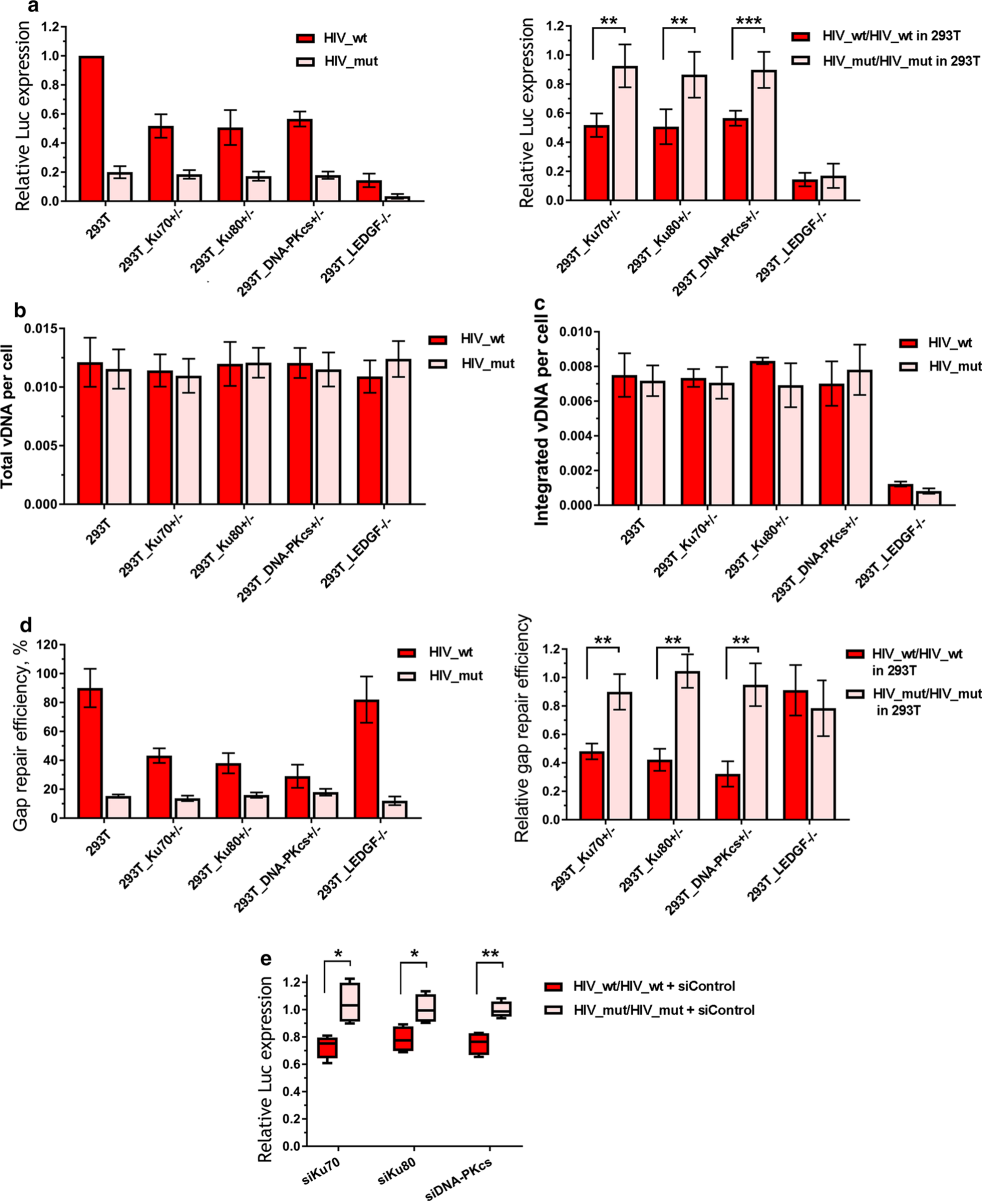
Distinct effects of KO of LEDGF/p75 or the components of DNA-PK on the early replicative stages of HIV. **a** Relative luciferase expression in 293T or different CRISPR/Cas9 modified cell lines transduced by 0.01 MOI of either HIV_wt or HIV_mut presented as: left plot—data normalized to the luciferase expression level in the parent 293T cell line transduced by HIV_wt; right plot—data normalized to the luciferase expression level in the 293T cells for HIV_wt and HIV_mut separately. **b**, **c** The level of total viral DNA (**b**) and integrated viral DNA (**c**) in 293T or different CRISPR/Cas9 modified cell lines transduced by either HIV_wt or HIV_mut measured by the standard qPCR analysis [45] and data was normalized to the data for CD3 gene to achieve a per cell normalization. **d** Gap repair efficiency in 293T or different CRISPR/Cas9 modified cell lines transduced by either HIV_wt or HIV_mut measured by the modified qPCR [39]. Data is shown as percentage of repaired proviral DNA relative to integrated proviral DNA (left plot) or as a repair efficiency of both pseudoviruses in CRISPR/Cas9 modified cell lines normalized to the repair efficiency in 293T cells (right plot) for HIV_wt and HIV_mut separately. **e** The siRNA mediated knockdown of Ku70, Ku80 or DNA-PKcs in 293T cells leads to a reduction in luciferase expression levels for HIV_wt and does not affect HIV_mut. Cells were transfected with each siRNA and 48 h later were transduced with 0.01 MOI of either HIV_wt or HIV_mut. 36 h later luciferase expression was measured and normalized to the luciferase levels in the control siRNA transfected cells for HIV_wt and HIV_mut separately. Means and SDs of (n = 3 for **a**–**d** and n = 4 for **e**) are plotted, significance was determined by two-tailed Student’s *t*-test, * = p < 0.05, ** = p < 0.01, *** = p < 0.001

Ku70 interacts with Ku80 and DNA-PKcs to form a DSB sensor in the NHEJ pathway. To examine whether these proteins are also involved in HIV-1 replication, we generated 293T Ku80^+/−^ and DNA-PKcs^+/−^ mKO cells using the CRISPR/Cas9-based knock-in–out approach described above for Ku70 (Additional file 1: Figure S8A and B). As it was the case for Ku70, mKO of either Ku80 or DNA-PKcs reduced the level of HIV_wt infectivity but did not affect the infectivity of HIV-1 with IN_mut (Fig. 4a), and this effect was also unrelated to transcription modulation (Additional file 1: Figure S4). Consistently, the infection of Ku80^+/−^ and DNA-PKcs^+/−^ cells with HIV_wt resulted in the decrease in I-R proviral DNA formation (Fig. 4d), but not in the production of either total or integrated viral DNAs (Fig. 4b, c) when compared to respective values obtained in the parental 293T cells. These data show that Ku80 and DNA-PKcs are both involved in HIV-1 replication at the step of post-integrational DNA repair.

**Fig. 5:**
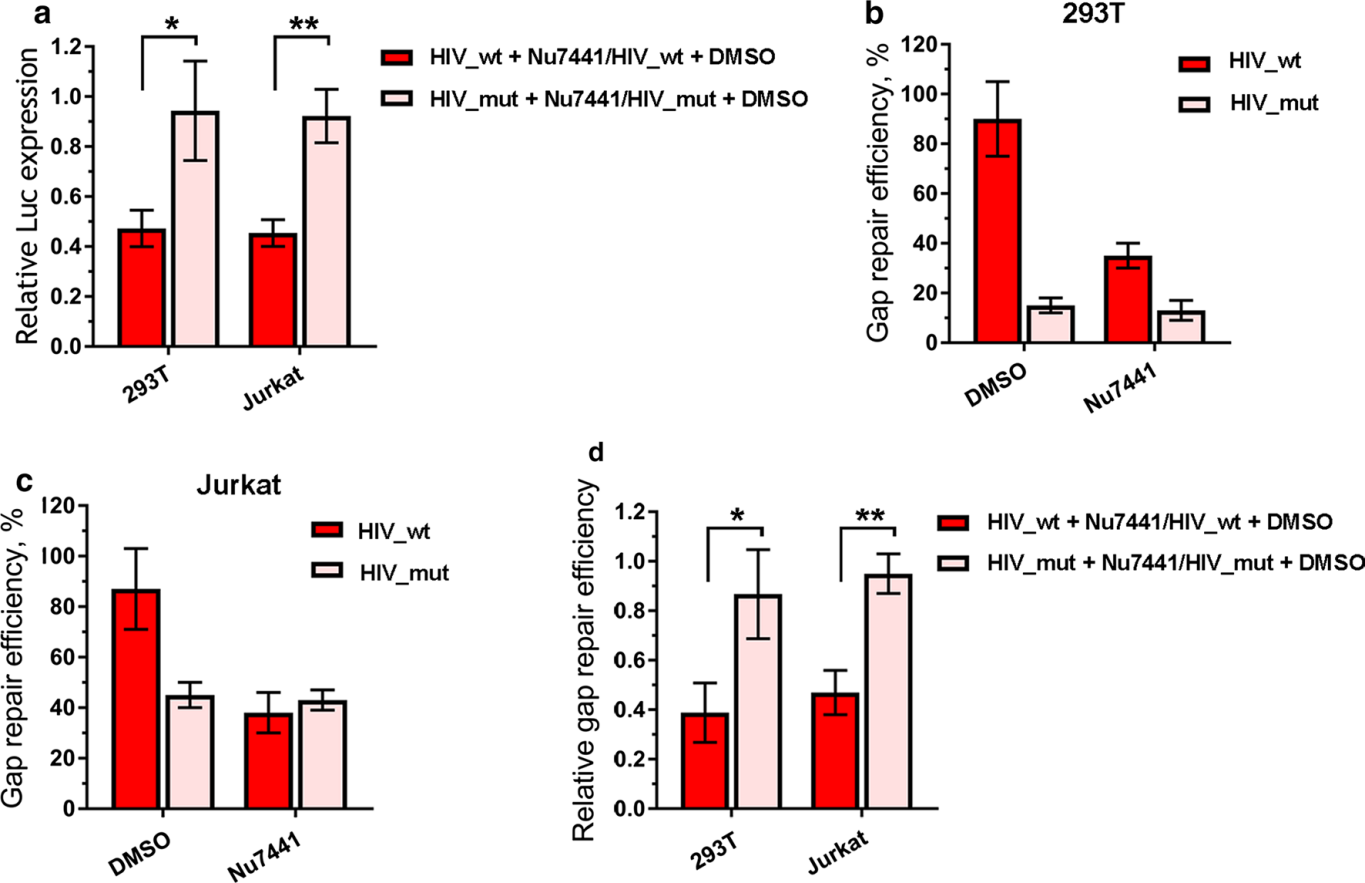
Effect of DNA-PKcs inhibitor Nu7441 on luciferase expression and post-integration gap repair for HIV_wt and HIV_mut. **a** Relative luciferase expression in 293T and Jurkat cells treated by DMSO or Nu7441 (1 µM for 293T and 5 µM for Jurkat) and transduced by 0.01 MOI of either HIV_wt or HIV_mut. Results are presented as data under Nu7441 treatment normalized to data under DMSO treatment for HIV_wt and HIV_mut separately. Mean values ± SD of n = 7 for 293T and n = 5 for Jurkat cells are presented. **b**, **c** Gap repair efficiency estimated for 293T (**b**) and Jurkat cells (**c**), data is presented as mean percentage of the total integrated proviral DNA ± SD (n = 5). **d** Relative gap repair efficiency presented as repair level in Nu7441 treated cells normalized to the level in DMSO-treated cells. Significance was determined by two-tailed Student’s *t*-test, * = p < 0.05, ** = p < 0.01

To further confirm the involvement of NHEJ pathway in post-integrational DNA repair, in addition to genetic manipulations we used a small molecule Nu7441, which specifically inhibits the DNA-PKcs catalytic activity [47]. The inhibitor and mock treated 293T or Jurkat cells were infected with either HIV_wt or HIV_mut, and the levels of infectivity and I-R proviral DNA formation were quantified as described above. The inhibition of DNA-PKcs activity resulted in the effects similar to those obtained with mKO of either of DNA-PK components. For example, the infectivity was decreased for HIV_wt, and no influence was observed on the transduction efficiency of HIV_mut (Fig. 5a). A qPCR-based analysis of the amount of I-R proviral DNA showed the same trend: Nu7441 affected the post-integrational gap repair only when cells were infected with HIV_wt, but not HIV_mut (Fig. 5b–d).

**Fig. 6:**
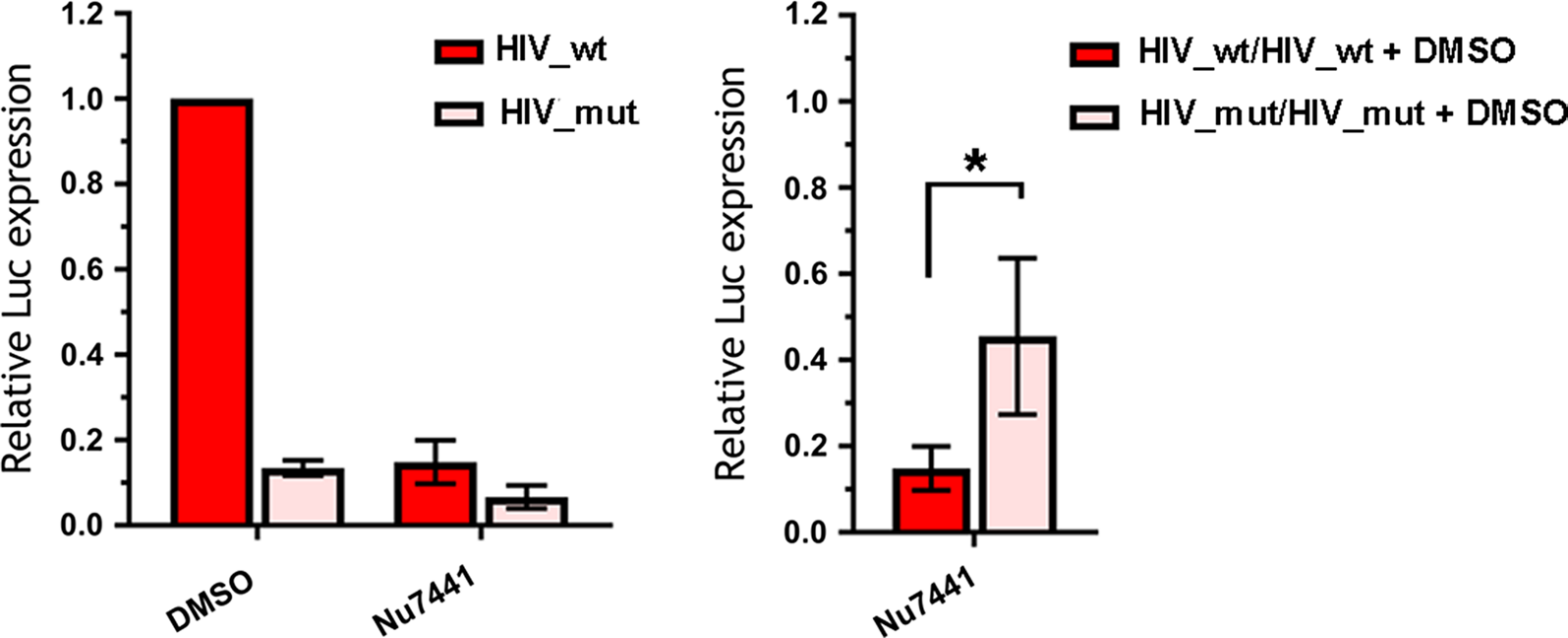
Effect of DNA-PKcs inhibitor Nu7441 on luciferase expression in primary human PBMCs. Cells were isolated from whole blood, activated and transductions were performed 48 h later with 0.1 MOI of either HIV_wt or HIV_mut in the presence of 7 µg/mL polybrene. Luciferase expression was assayed 48 h after transduction. Left plot: data is normalized to the luciferase expression level in DMSO-treated cells transduced by HIV_wt; right plot: luciferase expression level in Nu7441 treated cells is normalized to the luciferase level in DMSO-treated cells for each pseudovirus separately. Means and SDs of (n = 5) are presented. Significance was determined by two-tailed Student’s *t*-test, * = p < 0.05

When peripheral blood mononuclear cells (PBMCs) activated with phytogemagglutinin (PHA) were infected with HIV_wt or HIV_mut, the cell treatment with Nu7441 reduced the transduction efficiency by both variants of HIV-1 (Fig. 6, left histogram). However, the degree of inhibition was fourfold stronger for HIV_wt than for HIV-1 with IN_mut (Fig. 6, right histogram). These results clearly show that the phosphorylating activity of DNA-PKcs is required for the efficient HIV-1 post-integrational gap repair and viral infectivity in different cell lines and PBMCs.

Thus, we used genetic and biochemical approaches to demonstrate that along with Ku70, Ku80 and DNA-PKcs are also involved in HIV-1 post-integrational gap repair within the host DNA. This highlights the importance of NHEJ-mediated pathway of DNA repair for the efficient HIV-1 replication.

### Distinct effects of LEDGF/p75 and Ku70 knockout on HIV-1 infectivity and viral DNA product synthesis

To confirm the specificity of the influence of NHEJ pathway proteins on HIV-1 life cycle, we compared the impact of Ku70 mKO to that of the KO of LEDGF/p75, a well-known transcriptional co-activator, which interacts with HIV-1 IN and assists in viral integration by directing it into transcriptionally active chromosome units [3, 48, 49]. Using CRISPR/Cas9 method described for Ku70 mKO generation, we prepared 293T-LEDGF^−/−^ cell line with null phenotype (Additional file 1: Figure S8C). As expected, LEDGF/p75 KO dramatically reduced the level of HIV_wt infectivity (Fig. 4a, red bars), and a similar decrease of infectivity was also observed for HIV_mut (Fig. 4a, pink bars). The latter is not surprising since E212A/L213A mutation is out of the LEDGF/p75 binding site within IN [50]. However, in contrast to Ku70 mKO, LEDGF/p75 KO decreased the amount of integrated DNA in cells (Fig. 4c), but not the level of the I-R proviral DNA (Fig. 4d) that agrees with published data on the role of LEDGF/p75 in HIV-1 life cycle [3, 48, 49].

To rule out any suspicion that the HIV-1 replication changes observed in our work are caused by KO side effects, we evaluated HIV-1 infectivity and post-integrational DNA repair in 293T cells, where Ku70, Ku80 or DNA-PKcs were knocked down using small interfering RNAs (siRNAs) (Additional file 1: Figure S8D). The siRNA treated cells were infected with HIV_wt and HIV_mut and analyzed as described in previous paragraphs. Unlike control siRNA, all three specific siRNAs caused a significant decrease in the luciferase expression from HIV_wt, while no change was observed for HIV_mut (Fig. 4e). The observed effect was weaker than that for the CRISPR/Cas9 treated cells.

Therefore, we used several techniques for protein depletion and compared two cellular proteins, Ku70 and LEDGF/p75, which both interact with HIV-1 IN and are required for viral replication. We demonstrated that the NHEJ pathway of the host DNA repair system is specifically involved in the post-integrational gap repair step of HIV-1 life cycle.

### Amino acids of HIV-1 integrase involved in Ku70 binding have a low mutation rate

**Fig. 7:**
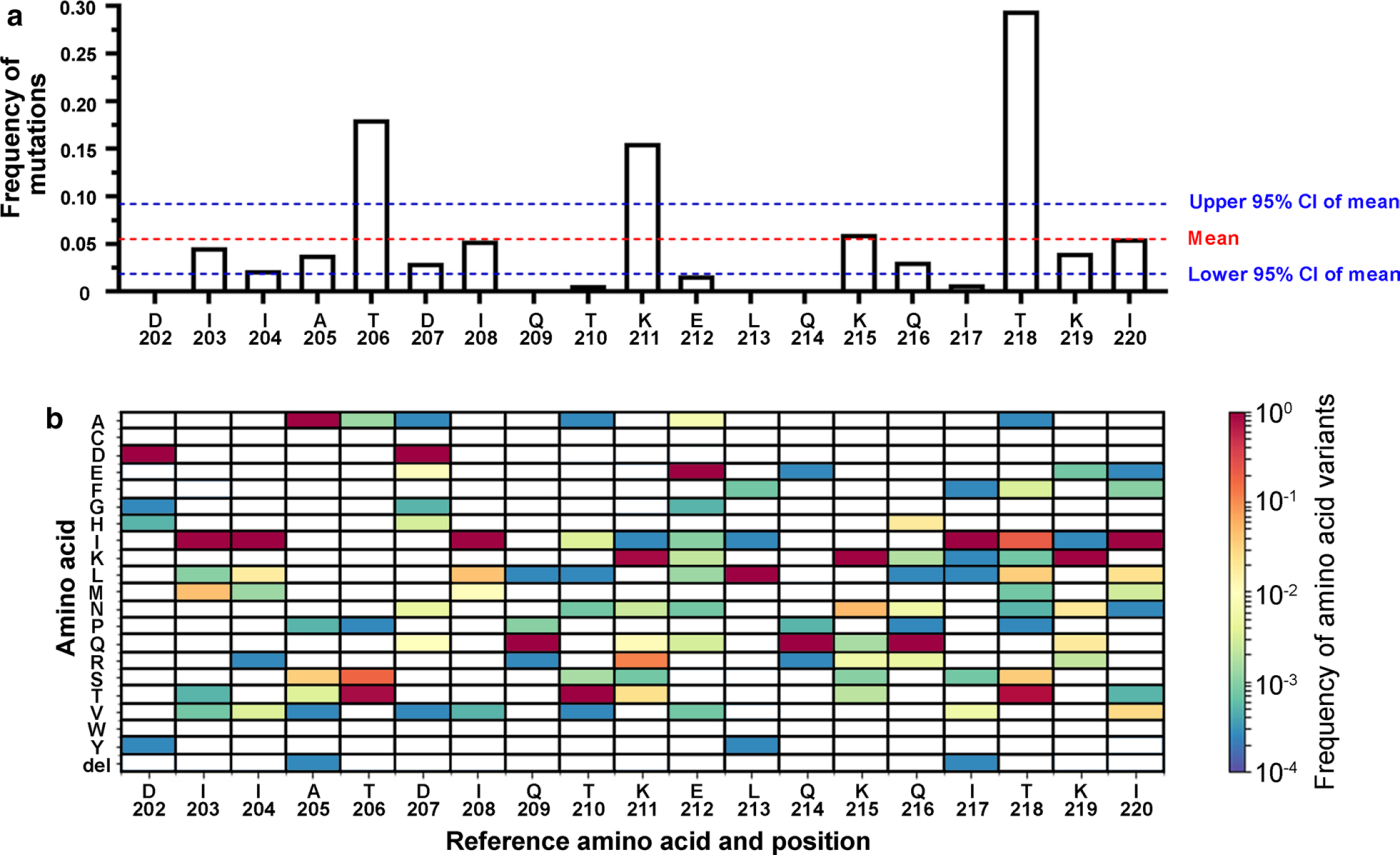
Mutation rates in α6-helix of HIV-1 integrase. Frequencies of mutations were calculated basing on 3862 sequences of *pol* gene deposited at http://www.hiv.lanl.gov/ and depicted as total mutation rate for X position (**a**) or as mutation matrix (**b**)

The HIV-1 reverse transcriptase lacks proofreading activity that results in extremely high spontaneous mutation level along the viral genome. However, the frequency of mutations differs for different positions, and the amino acid residues, which are important for the functioning of the virus, are less likely to be changed. The residues involved in Ku70 binding (E212 and L213) [33] are located in the region of α6-helix, which links C-terminal domain of HIV-1 IN and its catalytic core. We analyzed the frequency of mutations in α6-helix based on 3862 sequences of *pol* gene deposited at http://www.hiv.lanl.gov/ and identified that this region is relatively variable with a mean mutation rate equal to 0.05513 ± 0.03665 (mean ± 95% CI). However, the residues involved in Ku70 binding, E212 and especially L213, have lower mutation rates (0.0168 and 0.00129, respectively, Fig. 7). In contrast, mutation rates for residues K211, K215 and K219 that are dispensable for Ku70 binding [33] are higher (0.1560, 0.0606 and 0.0407 respectively against 0.00129 for L213, Fig. 7). Simultaneous substitution of both amino acids at 212/213 positions was observed only in 1 of 3862 sequences (frequency of mutation—0.000259), at 211/215 positions—in 33 of 3862 sequences (frequency of mutation—0.00854), at 211/219—in 42 of 3862 sequences (frequency of mutation—0.01088), at 215/219—in 9 of 3862 sequences (frequency of mutation—0.00233). In addition, we estimated frequency of mutations of amino acids A128, A129, W131 and W132, which are directly involved in LEDGF/p75 binding [50]. Their mutation rates were found to be equal to 0.00233 (for A128), 0.00155 (for A129), 0.00026 (for W131), and 0.00130 (for W132), and comparable with mutation frequency for L213 (0.00129). These results confirm once again the significance of the amino acid residues involved in the formation of the IN complex with Ku70 for HIV-1 replication.

## Discussion

The investigation of mechanisms of viral–host interactions during early steps of HIV-1 life cycle is important for the understanding of viral pathogenesis, and might also lead to the identification of new targets for antiretroviral therapy [51, 52]. The involvement of components of the NHEJ pathway in HIV-1 replication has been postulated in several studies [8, 20–23, 51–53], but the replication stage affected by NHEJ has not been determined. Nevertheless, depletion of DNA-PK components has been observed to lead to a higher level of cell death after HIV-1 infection [20, 21]. This finding made it possible to presume that the DNA-PK components are involved in post-integration DNA repair, and that the cell incapability of an efficient repair of the dsDNA breaks after viral DNA integration provides an apoptotic signal [20]. Here, we unambiguously show that the components of DNA-PK complex participate in the post-integrational DNA gap repair, describe the importance of interaction between IN and the cellular protein Ku70 for the gap repair step of HIV-1 life cycle and suggest a complex between these two proteins as a possible target for drug design.

By substituting E212 and L213 in HIV-1 IN to alanine, we showed earlier and here that these two amino acid residues are critical for maintaining interaction between viral IN and Ku70 (Fig. 2a and [33]) whereas having no influence on the intracellular stability of IN (Fig. 2b, c). A noticeable Ku70-induced stabilization of IN shown by us (Fig. 2b, Additional file 1: Fig. S1B) and others [25] could not be achieved through an interaction in the site that is formed by an intradomain α6-helix of IN and the N-terminal domain of Ku70 [33]. The stabilization might result from some interaction in a different site or by some other and less specific mechanism, e.g. deubiquitinating activity of Ku70 shown in several works [54, 55]. To ascertain whether IN interaction with Ku70 is important for HIV-1 replication, we constructed HIV-1 packaging vector bearing mutations in the IN gene coding for E212A/L213A substitutions and used one cycle replication assay to quantify a total infectivity of HIV-1 with wild type and mutant IN (HIV_wt and HIV_mut, respectively). We demonstrated that E212A/L213A double mutation in IN significantly reduces HIV-1 infectivity in different human cell lines (Fig. 3a). The importance of the complex formation between Ku70 and IN for HIV life cycle was further confirmed in experiments with genetic and siRNA-mediated knockdown, where the transduction by HIV_wt, but not HIV_mut was sensitive to Ku70 depletion (Figs. 3c and 4e).

We have earlier found that IN with E212A and L213A substitutions retains its catalytic activity in vitro although it is slightly decreased [33]. Here, we detected no differences between the levels of integrated proviral DNA in 293T cells infected either with HIV_wt or HIV_mut at low MOI (Fig. 3e), although at higher MOI, the HIV_mut showed a slight decrease in the amount of integrated proviral DNA (Additional file 1: Fig. S7C). Likewise, the amounts of total viral DNA measured in cells infected with HIV_wt and HIV_mut were similar (Fig. 3d). These results suggested that the binding of HIV-1 IN to Ku70 cellular protein is not essential for reverse transcription or integration. However, a qPCR protocol that is widely used to detect the total level of integrated proviral DNA [56] could not discriminate between repaired and non-repaired forms of proviral DNA. After modification of the protocol ([39] and Additional file 1: Figure S5), we found that E212A/L213A substitutions in IN resulted in a decreased amount of repaired proviral DNA in cells infected with HIV-1 24 h post transduction (Fig. 3f). The single-stranded DNA gaps should be repaired or they lead to the cell death under cell division process. Indeed, previously published data described HIV-1-mediated killing of normal murine lymphocytes in the presence of DNA-PKcs and ATM inhibitor—wortmannin [57] or in ATM-deficient cells [58]. We measured the levels of total and integrated viral DNA as well as of luciferase expression and gap repair efficiency 72 h post transduction (Additional file 1: Fig. S6). We did not detect any loss of proviruses, however, the levels of integrated-repaired viral DNA for both HIV_wt and HIV_mut proviruses were found to be equal. We assume that these facts may be explained by our experimental model: 293 cells constitutively express Ad5 E1A/E1B proteins [59] that deregulate pRB/p53 pathways [60], and as a result may lead to impaired cell death in response to DNA-damage.

Importantly, the decreased amount of repaired proviral DNA in cells infected with HIV-1 24 h post transduction was provided by mono-allelic KO of the Ku70 gene. Thus, we showed a relation between defects in HIV-1 infectivity associated with the viral IN inability to bind Ku70 or Ku70 depletion and the post-integrational gap repair efficiency. These data were further supported by our analysis of the frequency of mutations at positions 212 and 213 within HIV-1 IN. The mutation rate at these positions is very low (Fig. 7) and comparable with mutation rates for amino acids from the LEDGF/p75 binding pocket within IN. The conservancy of E212 and L213 suggests the biological importance of these amino acid residues in HIV-1 replication. In addition, it should be noted that E212A/L213A substitutions within IN lead to a defect in the viral replication, which significantly differs from the defects caused by the mutations described earlier [44–46]. Indeed, class 1 IN mutants are specifically blocked at the integration step and are typified by changes in the enzyme active site, and class 2 mutants induce defects at the step of viral particle assembly and/or reverse transcription. Here we have shown that both reverse transcription and integration are not influenced by E212A/L213A substitutions, and therefore this IN mutant might be considered as a “class 3”.

Ku70 in complex with Ku80 recognizes free DNA ends and binds DNA-PKcs to form a trimeric DNA-PK complex, which then triggers DNA DSB repair via NHEJ pathway [28–31]. To gain more evidence for the involvement of NHEJ-mediated repair in HIV life cycle, we generated 293T cells with Ku80 and DNA-PK mono-allelic KO and confirmed that all the effects of Ku70 on HIV-1 infectivity also manifested when either Ku80 or DNA-PKcs levels were reduced (Fig. 4). Therefore, we concluded that at least three major players of NHEJ-mediated DNA repair are involved in the post-integrational DNA repair step of HIV-1 life cycle.

After formation of the trimeric DNA-PK complex, DNA-PKcs catalyzes phosphorylation of downstream messengers [61], and we therefore assumed that inhibitors of the DNA-PKcs catalytic activity would affect HIV-1 replication. We proved this assumption using a selective inhibitor Nu7441, and showed that the addition of Nu7441 to 293T and Jurkat cell lines reduced the infectivity for HIV_wt but did not affect HIV_mut (Fig. 5a). In these experiments, an observed drop in luciferase expression was due to an impaired post-integrational gap repair (Fig. 5b, c). Finally, using the Nu7441 mediated DNA-PKcs inhibition, we verified our hypothesis on primary human blood cells (Fig. 6). Interestingly, the extent of inhibition was proportionally higher, when measured in activated PBMCs than in immortalized cell lines, tenfold vs twofold, respectively (Figs. 5a and 6). One possible explanation for such a difference could be that in immortalized cell lines other DNA repair pathways might additionally be involved in HIV-1 post-integrational gap repair [11–16].

Based on our study, we can propose a model where HIV-1 IN through direct interaction with Ku70 recruits a whole DNA-PK complex to the viral DNA integration site. DNA-PK then activates downstream mediators and initiates repair of the post-integrational gaps in proviral DNA. An efficient DNA repair enables HIV-1 to pass through viral life cycle. A key point in our model is the binding of IN with Ku70. Otherwise, the NHEJ-mediated gap repair would fail as Ku complex preferentially binds double-stranded DNA ends [62–64] that are absent during retroviral integration ([5, 9] and Fig. 1). To demonstrate an importance and specificity of the NHEJ system components for the DNA gap repair step completing HIV-1 integration, we compared the effects of Ku70 mKO and KO of LEDGF/p75, a well-known HIV-1 IN co-factor that assists in integration site selection and chromatin tethering [47, 65–68], on HIV-1 transduction efficiency. Unlike Ku70 and other NHEJ mediators, LEDGF/p75 KO had no influence on the level of post-integrational gap repair, but, expectedly, reduced the total level (not I-R fraction) of integrated proviral DNA (Fig. 4).

## Conclusions

We conclude that HIV-1 integrase recruits DNA-PK to the site of HIV post-integrational repair due to Ku70 binding—a novel finding, that explains the involvement of DNA-PK despite the absence of free double stranded DNA breaks. In addition, our data clearly indicate the importance of interactions between HIV-1 IN and Ku70 in HIV-1 replication at the post-integrational repair step. Based on these data, we assume that the complex of HIV-1 IN and human Ku70 can be viewed as a possible target for drug design.

## Methods

### Plasmids and oligonucleotides

pCDNA3_INhiv_HA, pCDNA3_INhiv_HAmut and pCDNA3_Ku70_3×FLAG vectors were described earlier [33]. To generate expression vectors for Ku80, Ku70 (1–250) and Ku70 (251–609), respective coding sequences were PCR-amplified from cDNA with primers indicated in Additional file 1: Table S1 and cloned into the pCDNA3_3×FLAG vector (Invitrogen) using NheI and AflII restriction sites. pCMVΔR8.2_mutIN coding HIV-1 IN with E212A/L213A double mutation was prepared using Quick Change II Site-Directed Mutagenesis Kit (Agilent Technologies, USA) based on pCMVΔR8.2 vector (Addgene plasmid #12263) using previously published primers IN_eu_212/213/IN_eu_212/213_anti [33]. pCMVΔR8.2_IN_E152A and pCMVΔR8.2_IN_H16C, pCMVΔR8.2_IN_F185A coding class-1 and class-2 IN mutants were prepared in the same way using primers IN_E152A/IN_E152A_anti, IN_H16C/IN_H16C_anti, IN_F185A/IN_F185A_anti (Additional file 1: Table S1). pGL3-CMV vector was obtained by PCR-amplification of CMV promoter from pCMVΔ8.2R plasmid with primers indicated in Additional file 1: Table S1 followed by cloning into the pGL3 vector (Promega) using BglII and HindIII cloning sites. pRL_hPGK cloning vector was constructed by substituting CMV promoter in pRL-CMV vector (Promega) with the human PGK promoter sequence, which was PCR-amplified (Additional file 1: Table S1, primers IX and X) and cloned into BglII and HindIII restriction sites.

### Cell cultures

293T cells were obtained through NIH AIDS Research and Reference Reagent Program. 293T cells were cultured in DMEM medium supplemented with 10% FBS and 100 I.U./mL penicillin/100 μg/mL streptomycin solution (all from Invitrogen). Jurkat, CEM (all purchased from ATCC) and PBMCs were cultured in RPMI medium supplemented with 10% FBS and 100 I.U./mL penicillin/100 μg/mL streptomycin solution (all from Invitrogen). PBMCs were isolated from whole healthy donor blood on a 1.077 g/cm^3^ Ficoll-Paque density gradient (PanEco, Russia), washed twice using PBS supplemented with 2% FBS and cultured in RPMI culture medium. Cells were activated with 1 µg/mL PHA (Sigma) for 2 days and grown in the presence of 100 U/mL of recombinant human interleukin-2 (Ronkoleikin, Biotech, Russia). The proportion of CD4+ cells in isolated PBMCs was equal to 63 ± 10% according to the flow cytometry analysis, performed on FACS Canto II (BD, USA) using APC-Cy7-conjugated anti-CD3 (clone SK7, BD Pharmingen, USA) and APC-conjugated anti-CD4 (clone RPA-T4, BD Pharmingen, USA) antibodies. All experiments with the human blood samples were approved by the Human Ethics Committee of the Institute of Immunology (Moscow), and blood donors gave informed consent for the use of their samples in the described experiments. All methods were performed in accordance with relevant the guidelines and regulations.

### Transfections and infections

For a plasmid DNA, 293T cells were transfected for 6 h with TurboFect transfection reagent (Thermo Scientific) in accordance with manufacturer’s instruction. To transfect siRNAs, 50 pmol of pre-annealed siRNA duplex in OptiMem transfection medium were mixed with Lipofectamine RNAiMAX reagent (Invitrogen) according to manufacturer’s protocol and then added to 293T cells seeded in a 12-well plate in 1 mL of growth medium for 6 h. All siRNAs used in this work are listed in Additional file 1: Table S2. For Dual-Luciferase assay 30 ng of pGL3_CMV vector was transfected into 1 × 10^5^ 293T cells as indicated above together with 10 ng of pRL_hPGK vector. At 48 h post-transfection, cells were lysed and analyzed with Dual Luciferase Reporter Assay system (Promega) and VICTOR X Multilabel Plate Reader (Perkin Elmer).

To generate HIV-like pseudoviruses, 293T cells were co-transfected with HIV-1 packaging vector pCMVΔR8.2 (Addgene plasmid #12263) or pCMVΔR8.2_mutIN, vector for expression of protein G from vesicular stomatitis virus (VSV) pCMV-VSVG (Addgene plasmid #8454), and reporter plasmid pUCHR-inLuc-mR [69, 70]. Forty-eight hours post-transfection supernatants were harvested, pseudoviruses were concentrated by centrifugation at 30,000×*g* for 2 h and resuspended in PBS. The level of p24 was assayed using the HIV-1 p24-antigen ELISA Kit (Vector Best, Russia). 293T cells were infected by the addition of pseudoviruses to the cell media at final concentration of 10 pg of p24 per 10^5^ cells. In case of experiment with HIV_E152A, HIV_H16C or HIV_F185A we infected cells by the addition of wild type or mutated pseudoviruses to the cell media at final concentration of 1.3 ng of p24 per 10^5^ cells. MOI was calculated as amount of total (reverse transcribed) viral DNA per cell in case of HIV_wt transduced cells. To enhance transduction capability of pseudoviruses, the Jurkat, CEM and PBMCs were transduced by spinoculation at room temperature and 2000×*g* for 1.5 h in the presence of 7 µg/mL of polybrene (Sigma). Cells were harvested at 24 h or 48 h post infection, cell number was counted, and Luciferase activity in cell lysates was measured using Victor X5 2030 (Perkin Elmer) plate reader and Luciferase assay system kit (Promega). The resulting data was normalized to cell count.

Integrase stabilization experiments (Fig. 2b and Additional file 1: Fig. S1B) were carried out as following: 2 × 10^5^ cells were co-transfected with 0.75 μg of pCDNA3_IN_HA or pCDNA3_INmut_HA vectors and 1.5 μg of empty pCDNA3.1, pCDNA3_Ku70_3×FLAG or pCDNA3_Ku80_3×FLAG vectors. 48 h after transfection cells were lysed and the analysis of HA-tagged integrase, Flag-tagged Ku70 or Ku80 and tubulin amounts was performed by Western-blot.

### Generation of knockout cells with CRISPR/Cas9

Guide RNA (gRNA) protospacer sequences were selected using two web-based resources http://crispr.mit.edu/ and http://chopchop.cbu.uib.no/ and cloned into pKS gRNA BB plasmid [71] using BbsI restriction site. Sequences are listed in Additional file 1: Table S3. The gRNA target sequences for the human Ku70, Ku80, DNA-PK and LEDGF/p75 were the following: ATGTAGTGCCATTCGGTGTG, CCGGCAACATGGTGCGGTCG, TTGTCCGCTGCGGACCGCTG, and TGTTTCGGGGGCGAGACCGG, respectively. The ~ 100 nt homology arms in a close proximity to DNA cut sites were included into synthetic oligonucleotides together with ~ 18 nt sequences of complementarity to the plasmid templates (Additional file 1: Table S4). Donor DNAs containing short ORF comprised of HA-tag embedded into CD52 and transcriptional terminator from the human β-globin were PCR-amplified from either pJet-CMV-CD5HA2-bglpA plasmid (for K70, Ku80, DNA-PK) or pUCHR-mClover-smAID-P2A-CD5HA2-bglpA plasmid (for LEDGF/p75) [42]. PCR products were gel purified and used to co-transfect cells with CRISPR/Cas9 components. Targeted knock-in of CD5HA2 with concomitant gene disruption was achieved by co-transfecting 293T cells with the spCas9 expression plasmid (Addgene), the gRNA coding plasmid and a respective PCR-donor. Three days post transfection, HA^+^ cells were sorted out after immunofluorescent staining with rabbit anti-HA mAb clone C29F4 (Cell Signaling Technology, USA) using FACSAria II Instrument (Becton–Dickinson Biosciences, San Jose, CA, USA). The expression of target gene was estimated by WB as outlined below. Since homozygous KO of Ku70, Ku80 and DNA-PK was lethal for cells, we were able to grow only monoallelic KO cells with reduced expression of target gene. In contrast, LEDGF/p75 KO cells selected via HA expression carried null phenotype, as this transcriptional co-activator is dispensable for cell viability [65, 66].

### qPCR

To quantify mRNA level, a total RNA from 1 × 10^6^ cells was isolated using Trizol reagent (Invitrogen). cDNA was synthetized using the MMLV kit from Evrogen (Russia), and qPCR was performed with primers indicated in Additional file 1: Table S1 using the qPCR Mix-HS SYBR from Evrogen (Russia) on a Biorad CFX96 amplifier (Biorad). The total and integrated vDNA were quantified 24 h.p.i., unless otherwise indicated, as previously described [56]. Post-integrational gap repair efficiency was measured by modified Alu-specific PCR as described in detail in [39].

### Co-immunoprecipitation

293T cells (4 × 10^6^) in 25 cm^2^ flasks were transfected with 9 μg of empty pCDNA3.1 vector (negative control) or co-transfected with 2 μg of pCDNA3_Ku70_3×FLAG and 7 μg of pCDNA3_IN_HA/pCDNA3_INmut_HA/pCDNA3.1 vectors as described above. 48 h after transfection cells were lysed for 30 min on ice in RPMI medium (Invitrogen) supplemented with Protease inhibitor cocktail (Thermo Scientific) and 0.25% NP-40 (Helicon). Lysates were cleared by centrifugation for 10 min at 14,000×*g* and protein concentration was measured on NanoDrop 2000 spectrophotometer (Thermo Scientific). 0.1 mg of cell lysates were saved for input analysis. 1 mg of total protein was mixed with HA-antibody conjugated agarose (Sigma) or FLAG-antibody conjugated agarose (Sigma) and incubated for 4 h at 4 °C. Beads were washed 4 times with lysis buffer and bound proteins were eluted with 0.1 M glycine pH 2.5 for 20 min at room temperature. Elution fractions and inputs were then analyzed by Western blot.

### Western blot

Cells were washed with ice-cold PBS, pelleted and lysed in 20 mM Tris–HCl pH 7.5, 150 mM NaCl, 0.5% Np-40 supplemented with Protease inhibitor cocktail (Thermo Fisher Scientific) on ice for 30 min and lysates were cleared by centrifugation for 10 min at 14,000×*g*. Total protein concentration was measured by Bradford assay and 10–50 µg of protein was mixed with loading buffer. For the analysis of IN, Ku70, Ku80 and LEDGF, protein samples were separated on 12% (IN) or 10% (Ku and LEDGF) SDS PAGE and transferred to Immun-Blot PVDF Membrane (Bio-Rad) in buffer containing 50 mM Tris–HCl pH 7.5, 40 mM glycine, 20% ethanol, 0.08% SDS. For the analysis of DNA-PKcs, cell lysates were mixed with 1× protein loading buffer containing 20 mM Tris–acetate, pH 7.2, 0.1% SDS, 0.01 M DTT, ran on 6% PAGE with 20 mM Tris–acetate buffer, pH 7.2 and transferred to PVDF membrane in 25 mM tricine, 25 mM Tris–acetate buffer, pH 7.2 with 1 mM EDTA, 10% ethanol and 0,01% SDS. The primary antibodies used were: anti-Ku70 rabbit polyclonal antibody (ab83502, Abcam), anti-Ku80 rabbit polyclonal antibody (#2735, Cell Signaling), anti-PRKDC (DNA-PKcs) rabbit antibody (SAB4300443, Sigma), anti-NONO rabbit antibody (N8664, Sigma), anti-PSIP1 (LEDGF) mouse monoclonal antibody (sc-101087, Santa Cruz Biotechnology), anti-FLAG M2 HRP-conjugated antibody (A8592, Sigma), anti-actin rabbit N-terminal antibody (A2103, Sigma), mouse anti-human tubulin clone 12G10 mAb (Developmental Studies Hybridoma Bank at the University of Iowa). To detect IN, an anti-integrase rabbit serum was used (a kind gift from Dr. M. Isaguliants, Karolinska Institutet, Sweden). HRP-conjugated anti-rabbit (Sigma) and anti-mouse antibodies (Sigma) were used as secondary antibodies. Immuno-reactive bands were detected on ChemiDoc MP system (Bio-Rad) using Clarity Western ECL substrate (Bio-Rad).

### Cycloheximide protein stability assay

2 × 10^5^ cells were co-transfected with 1.5 μg of pCDNA3_IN_HA or pCDNA3_INmut_HA vectors and 0.75 μg of empty pCDNA3.1 or pCDNA3_Ku70_3×FLAG vectors as described above. 48 h after transfection cells were treated by 50 μg/mL cycloheximide (Cell Signaling) for 0; 20; 40; 60; 120 or 180 min with subsequent lysis and analysis of HA-tagged integrase and tubulin amount by Western-blot.

### Sequence analysis

Protein alignment of the polypeptide coded by HIV-1 *pol* gene was obtained from HIV sequence database http://www.hiv.lanl.gov/. The latest available collection (2017) has 4819 sequences. After filtering out sequences with uncanonical symbols (# or X symbols) the remaining set had 3862 sequences. The frequency of occurrence for each individual amino acid was calculated and plotted with Matplotlib with uniprot sequence Q76353 as a reference.

## Supplementary information

**Additional file 1.** Additional materials and methods, Supplementary tables S1–S4 and Supplementary figures S1–S8.

## Data Availability

The data for this study is available from the corresponding author on reasonable request.

## Abbreviations

HIV-1: human immunodeficiency virus type 1
NHEJ: non-homologous end joining
IN: integrase
KO: knockout
HR: homologous recombination
BER: base excision repair
DSB: double strand break
DNA-PKcs: catalytic subunit of DNA-dependent protein kinase
IN_wt: wild type integrase
IN_mut: integrase bearing substitutions E212A/L213A
HIV_wt: HIV-based pseudoviruses containing native IN
HIV_mut: HIV-based pseudoviruses containing IN_mut
HIV_E152A: HIV-based pseudoviruses containing integrase bearing substitution E152A
HIV_H16C: HIV-based pseudoviruses containing integrase bearing substitution H16C
HIV_F185A: HIV-based pseudoviruses containing integrase bearing substitution F185A
MOI: multiplicity of infection
SORTS: Surface Oligopeptide knock-in for Rapid Target Selection
mKO: monoallelic gene knockout
PBMCs: peripheral blood mononuclear cells
siRNAs: small interfering RNAs
