# Supplementary material for: NHEJ pathway is involved in post-integrational DNA repair due to Ku70 binding to HIV-1 integrase
Source: Retrovirology. 2019 Nov 6;16:30. doi: 10.1186/s12977-019-0492-z (PMC6833283; doi:10.1186/s12977-019-0492-z)
Supplement: Supplementary file 1 — Additional file 1. Additional materials and methods, Supplementary tables S1–S4 and Supplementary figures S1–S8. [file 12977_2019_492_MOESM1_ESM.docx]

**NHEJ pathway is involved in post-integrational DNA repair due to Ku70 binding to HIV-1 integrase**

Ekaterina Knyazhanskaya^1,2†^ & Andrey Anisenko^1,2†^*, Olga Shadrina^3^, Anastasia Kalinina^4^, Timofei Zatsepin^1,5^, Arthur Zalevsky^3^, Dmitriy Mazurov^6,7^, Marina Gottikh^[[1]](#footnote-1),2^

**Additional materials and methods**

*Post-integrational gap repair efficiency quantification:*

1. To quantitatively estimate the gap repair efficiency, 200-400 ng of total DNA obtained from infected cells are divided between two PCR tubes.
2. Into tube 1 (signed as “rep”) 150 nM ULF1 primer is added, as well as 1X DreamTaq buffer, 1.25 U of DreamTaq polymerase, dNTP mix (0.4 mM of each) and 1 mM MgCl_2_. Total volume = 20 μL.
3. The linear pre-amplification (LPA) cycling conditions:

Step 1: 95^0^C 8 min

Step 2: 95^0^C 1 min

Step 3: 55^0^C 1 min

Step 4: 72^0^C 10 min

Number of cycles (steps 2-4): 24

Step 5: 72^0^C 15 min

1. After finishing the LPA stage 5 μL of 1X DreamTaq buffer with 300 nM of each Alu1, Alu2, HCD3OUT3’, and HCD3OUT5’ primers, dNTP mix (0.4 mM of each) and 1 mM MgCl_2_ are added to the same tube.
2. The exponential pre-amplification (EPA) cycling conditions:

Step 1: 95^0^C 3 min

Step 2: 95^0^C 1 min

Step 3: 55^0^C 1 min

Step 4: 72^0^C 10 min

Number of cycles (steps 2-4) 12

Step 5: 72^0^C 15 min

Note: Only 12 cycles of amplification were performed to remain in the exponential phase.

1. Into tube 2 (signed as “int” ) 150 nM ULF1 should be added together with 300 nM of each Alu1, Alu2, HCD3OUT3’, and HCD3OUT5’ primers, 1X DreamTaq buffer, 1.25 U of DreamTaq polymerase, dNTP mix (0.4 mM of each) and 1 mM MgCl_2_. Total volume = 25 μL. The EPA procedure is performed as described in 5.
2. The TaqMan PCR is carried out on a Biorad CFX96 instrument (Biorad) with the Taq DNA-polymerase (Thermo) following the manufacturer's instructions. All reactions (for “repaired” HIV DNA, integrated HIV DNA, and CD3) are performed in a final volume of 20 μL containing 5 μL of a 1/5 dilution of the PCR products obtained in EPA stage. The appropriate sets of primers (250 nM Lambda T and UR2 for “repaired” and integrated HIV DNA, and HCD3IN5′ and HCD3IN5′ for the CD3 gene) are added to the master mix. The UHIV TaqMan probe (200 nM) was added to tube 1 (“rep”) and tube 2 (“int”) reaction mixtures. For CD3 quantification, 200 nM of the CD3 TaqMan probe is used. The same amplification steps are used for all reactions: a denaturation step (95°C for 4 min), followed by 40 cycles of amplification (95°C for 10 s, 60°C for 20 s).
3. For each sample four C_t_ values are obtained: C_t, rep_ (integrated viral DNA with linear pre-amplification) C_t, CD3 for rep_, C_t, int_ (integrated viral DNA without linear pre-amplification), and C_t, CD3 for int_. Then, C_t, rep_ and C_t, int_ are normalized to the amount of CD3 gene in the same sample.

C’_t, rep_= C_t, rep_- C_t, CD3 for rep_ and C’_t, int_= C_t, int_- C_t, CD3 for int_.

Find ΔC_t_ = C’_t, int_ - C’_t, rep_

Using formula $n=\frac{1}{1-\frac{2^{{\Delta C}_{t}}-1}{\alpha}}-1$ find repair efficiency from ΔC_t_ value.

**Additional tables**

**Table S1. Oligonucleotides used for preparation of expression vectors and as primers for qPCR.**

|  | Name | Oligonucleotide sequence (5’-3’) |
| --- | --- | --- |
| I | Ku70(250)_fw | gtacgctagcatgtcagggtgggagtcatattac |
| II | Ku70(250)_rv | ccaacttaagggttcgcgccaaggag |
| III | Ku70(609)_fw | gtacgctagcatgaccaggaagcgagcact |
| IV | Ku70_Cterm | ccaacttaagGTCCTGGAAGTGCTTGGT |
| V | Ku80_Nterm | gtacgctagcATGGTGCGGTCGGGGAATAA |
| VI | Ku80_Cterm | ccaacttaagtatcatgtccaataaatcgtccac |
| VII | Fw_CMVpr_pGL | gtacAGATCTgtgatgcggttttggcag |
| VIII | Rw_CMVpr_pGL | gtacAAGCTTagctctgcttatatagacctccc |
| IX | Fw_PGK_pRL | gtacAGATCTggggttggggttgcgc |
| X | Rw_PGK_pGL | gtacAAGCTTctggggagagaggtcgg |
| XI | Ku70_352_fw | tctgcctagcgataccaagag |
| XII | Ku70_352_rv | caacggcttgaaacccatga |
| XIII | Ku80_1_fw | GGACGTGGGCTTTACCATGA |
| XIV | Ku80_1_rv | TGCCATCTGTACCAAACAGGA |
| XV | GAPDH_fw | CAACAGCACACCCACTCCT |
| XVI | GAPDH_rv | ACCCTGTTGCTGTAGCCA |
| XVII | ULF1 | ATGCCACGTAAGCGAAACTCTGGGTCTCTCTGGTTAGAC |
| XVIII | Alu1 | TCCCAGCTACTGGGGAGGCTGAGG |
| XIX | Alu2 | GCCTCCCAAAGTGCTGGGATTACAG |
| XX | UR1 | CCATCTCTCTCCTTCTAGC |
| XXI | LambdaT | ATGCCACGTAAGCGAAACT |
| XXII | UR2 | CTGAGGGATCTCTAGTTACC |
| XXIII | HCD3OUT3’ | ACTGACATGGAACAGGGGAAG |
| XXIV | HCD3OUT5’ | CCAGCTCTGAAGTAGGGAACATAT |
| XXV | HCD3IN5’ | GGCTATCATTCTTCTTCAAGGT |
| XXVI | HCD3IN3’ | CCTCTCTTCAGCCATTTAAGTA |
| XXVII | UHIV_TaqMan | FAM-GCACTCAAGGCAAGCTTTATTGAGG-BHQ-1 |
| XXIX | CD3_TaqMan | FAM-AGCAGAGAACAGTTAAGAGCCTCCAT-BHQ-1 |
| XXX | IN_E152A | attttctttaattctttattcatagatgctattactccttgactttggggattg |
| XXXI | IN_E152A_anti | caatccccaaagtcaaggagtaatagcatctatgaataaagaattaaagaaaat |
| XXXII | IN_H16C | TGCTCTCCAATTACTGCAATATTTCTCATGTTCTTCTTGGGCCTTATC |
| XXXIII | IN_H16C_anti | GATAAGGCCCAAGAAGAACATGAGAAATATTGCAGTAATTGGAGAGCA |
| XXXIV | IN_F185A | CCCAATCCCCCCTTTTCTTTTAGCATTGTGGATGAATACTGCCAT T |
| XXXV | IN_F185A_anti | AATGGCAGTATTCATCCACAATGCTAAAAGAAAAGGGGG GATTGGG |

**Table S2. RNA oligonucleotides used as siRNAs in the work.**

|  | Name | Oligoribonucleotide sequence (5’-3’) |
| --- | --- | --- |
| rI | siControl_s | AGGUCGAACUACGGGUCAAdTdC |
| rII | siControl_as | UUGACCCGUAGUUCGACCUdAdG |
| rIII | siKu70_352_s | GUGCAAAACGAAUUCUAGAdTsdT |
| rIV | siKu70_352_as | UCUAGAAUUCGUUUUGCACdTsdT |
| rV | siKu70_1025_s | GCUAAAACGGUUUGAUGAUdTsdT |
| rVI | siKu70_1025_as | AUCAUCAAACCGUUUUAGCdTsdT |
| rVII | siKu80_179_s | ACAAGGAUGAGAUUGCUUUdTsdT |
| rVIII | siKu80_179_as | AAAGCAAUCUCAUCCUUGUdTsdT |
| rIX | siKu80_423_s | CAUGGGAAAUCAAGUUCUAdTsdT |
| rX | siKu80_423_as | UAGAACUUGAUUUCCCAUGdTsdT |
| rXI | siKu70_DNA-PK_s | СUAUGAAACUACUGAAGGAdTsdT |
| rXII | siKu70_DNA-PK_as | UCCUUCAGUAGUUUCAUAGdTsdT |

**Table S3. DNA oligonucleotides for generation of gRNA expression plasmids.**

|  | Name | Oligonucleotide sequence (5’-3’) |
| --- | --- | --- |
| I | 5’-gR-Ku70int | CACCGATGTAGTGCCATTCGGTGTG |
| II | 3’-gR-Ku70int | AAACCACACCGAATGGCACTACATC |
| III | 5’-gR-Ku80 | CACCGCCGGCAACATGGTGCGGTCG |
| IV | 3’-gR-Ku80 | AAACCGACCGCACCATGTTGCCGGC |
| V | 5’-gR-DNA-PK | CACCGTTGTCCGCTGCGGACCGCTG |
| VI | 3’-gR-DNA-PK | AAACCAGCGGTCCGCAGCGGACAAC |
| VII | 5-gR-LEDGF-95 | CACCGTGTTTCGGGGGCGAGACCGG |
| VIII | 3-gR-LEDGF-95 | AAACCCGGTCTCGCCCCCGAAACAC |

**Table S4. Sequences of primers to generate donor DNA using PCR.** Sequences, which are complementary to the plasmid templates are shown in bold.

|  | Name | Oligonucleotide sequence (5’-3’) |
| --- | --- | --- |
| I | 5’-Ku70-in | CTTTCGCCTAGTGAGCAGTAGCCAACTTGTCAGGGTGGGAGTCATATTACAAAACCGAGGGCGTTGAAGAAGCAGAGGAAGAACAAGAAGAGAACCTT**GCAGGTCGACCACCATG** |
| II | 3’-Ku70-in | GGAAGAAAAGAATTTCAGAATTATCTGGGAGGAGAAGGGCAGGCCATCAAACAGCAAAGTCCATCCAGCAGAGATAGGCAGGGAAGGTCTTTCTT**CCACACAAAAAACCAACACAC** |
| III | 5’-Ku80-in | CGAAGCGGCTCTTTCCGCTATCTGCCGCTTGTCCACCGGAAGCGAGTTGCGACACGGCAGGTTCCCGCCCGGAAGAAGCGACCAAAGCGCCTGAGGACCG**GCAGGTCGACCACCATG** |
| IV | 3’-Ku80-in | CTTTGTCTTGACCGCGATCCCACGATTCCTGCTTCCGAACCACCCTCTCCGGATCCCCAGTCCGGGTAAAGCCCAAGTCCATGGCTTTCTTTATA**CCACACAAAAAACCAACACAC** |
| V | 5’-DNA-PK-in | GCCGAGCGGGCGCACGCGCGGGAGCGGGACTCGGCGGCTTGGCGGGCTCCGGAGCCGGTGTGCGTTGCTCCCTGCTGCGGCTGCAGGAGACCTTGTCC**GCAGGTCGACCACCatg** |
| VI | 3’-DNA-PK-in | CCCGCGGCCCAGCTCGGGCCGGTACCCACCCAGCACCGCGGGGCTGCTGCTCAGGACGCATTCCTGCCCCAGGCCGCGGATCAGTTGATGACCGG**CCACACAAAAAACCAACACAC** |
| VII | 5-LEDGF-in-2 | ggcatcctcccccgcccgcgggcccggtagctgggcccgcgtccgccgcccgcatccccgcgccgccgcatctcctcgccgcctcccgggcttcgg**accACCATGAAGCGCTTCC** |
| VIII | 3-LEDGF-in-2 | CTGCTAAGCGCGAGGGCTACAAATCACTTACTCGAGCTGGCCAATGGGGATAACCTTTCATCTTGGCGAAGATGAGGTCTCCAGGTTTGAAATCG**CACACAAAAAACCAACACAC** |

**Additional Figures**


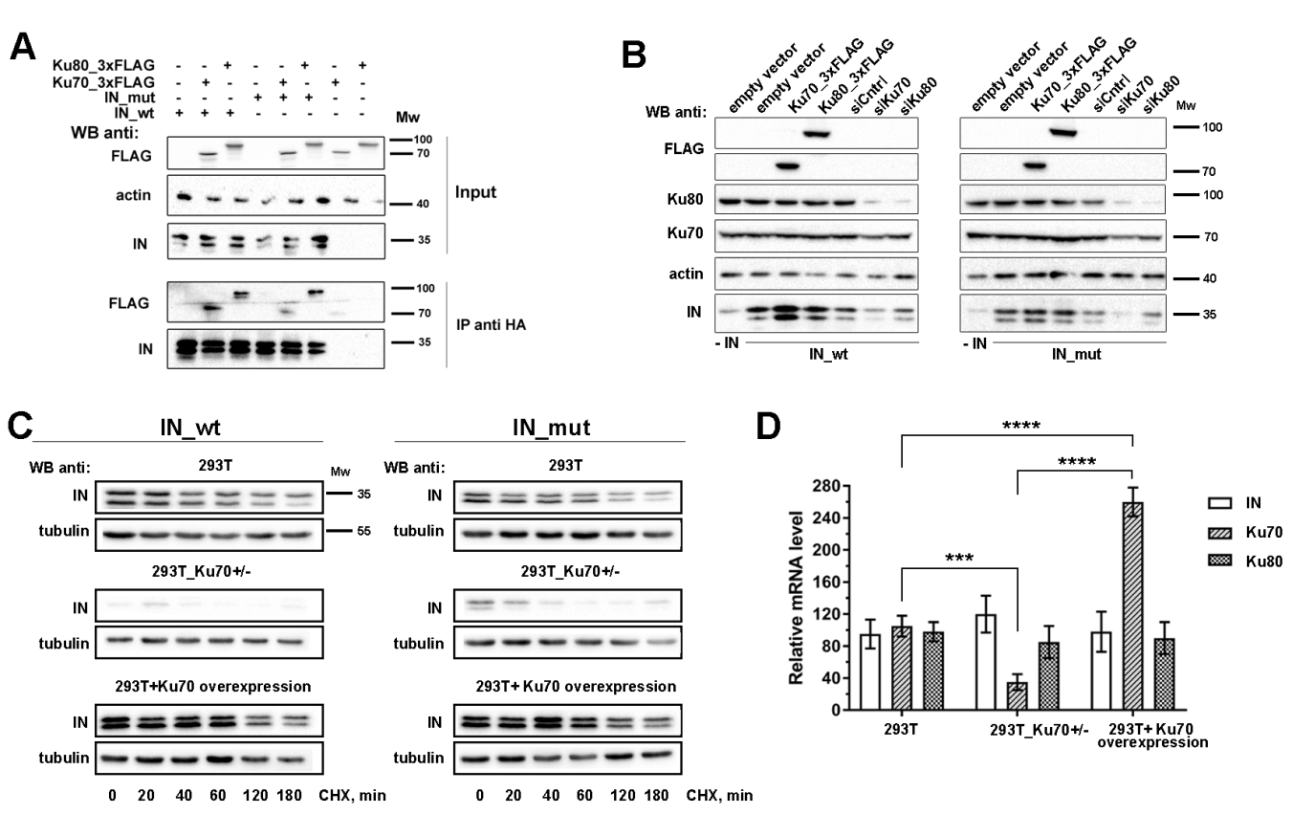


**Figure S1. Mutant integrase interacts with Ku80 at similar levels as the wild type integrase. Effect of different intracellular Ku70 concentrations on IN mRNA and protein stability.**

**(A)** Immunoprecipitation of HIV-1 IN_HA on anti-HA antibody conjugated beads (lower panel). IN_wt or IN_mut were expressed in 293T cells with Ku80_3xFLAG or with an empty vector, then cells were lysed, 10% of lysates were saved for input analysis (upper panel), the rest was subjected to immunoprecipitation for 4h at 4 °C, then the beads were washed 4 times with incubation buffer and proteins were eluted by 0.1M glycine, pH 2.5, and the eluates together with input samples were analyzed by Western blot. Representative images of at least three independent experiments are shown. **(B)** Western blot analysis of a superexpression of IN_wt (left panel) or IN_mut (right panel) either in the presence of superexpressed Ku70_3xFLAG, Ku80_3xFLAG or siRNA for Ku70 or Ku80. Representative image of at least three independent experiments is shown. **(C)** IN dynamic stability upon cycloheximide inhibition in 293T parent cell line (upper and lower panels) and 293T-Ku70+/- cells (middle panel) transiently transfected by WT or E212A/L213A mutant integrase expressing vectors under standard conditions (upper panel), Ku70 stable downregulation (middle panel), or Ku70 overexpression (lower panel). (**D)** Relative mRNA level for Ku70, Ku80 and HIV-1 IN_wt under the same conditions. Means and SDs of (n=3) are plotted, significance was determined by two-way ANOVA, *** = p<0.001, **** = p<0.0001. The weight marker migration positions are presented to the right of the WB panels.

**
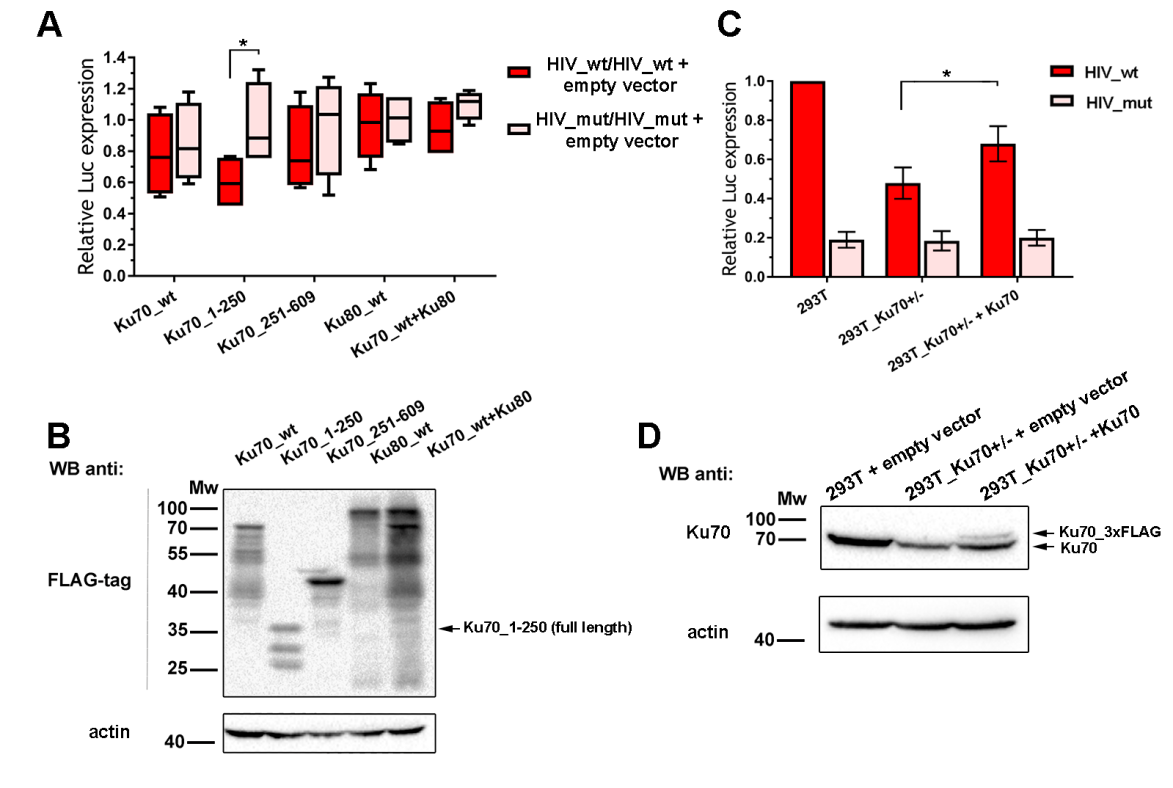
**

**Figure S2. Ku70 N-terminal domain interferes with HIV_wt replication presumably by competing with endogenous Ku70 for binding with IN_wt and not with IN_mut defective in Ku70 binding.**

(**A)** Relative luciferase expression of WT and mutant vectors in cells, transfected by an empty plasmid vector, or by plasmids expressing full length Ku70, Ku70 N-terminal domain (a.a. 1-250), Ku70 DNA-binding and C-terminal domains (a.a. 251-609), Ku80 or Ku70 + Ku80. The data is presented as normalized to the luciferase expression in mock transfected cells for each viral vector separately. (**B)** Western blot of cell lysates described in A, showing the expression levels of Ku70, its deletion mutants, Ku80 and Ku70 + Ku80 with actin as a loading control. All recombinant proteins are FLAG-tagged. A representative image of three independent experiments is shown. The weight marker migration positions are presented to the left of the WB panels. **(C)**. Effect of Ku70 overexpression in 293T_Ku70+/- cells on luciferase expression under HIV_wt or HIV_mut transduction. 293T-Ku70+/- cells 48 hours before transdution were transfected with Ku70_3xFLAG or with an empty vector, 293T cells were transfected with empty vector. **(D)** Western blot analysis of Ku70 levels for cell lysates obtained in (C). Means and SDs of (n=3) are plotted. Significance was determined by two-tailed Student’s *t*-test, * = p<0.05.

**
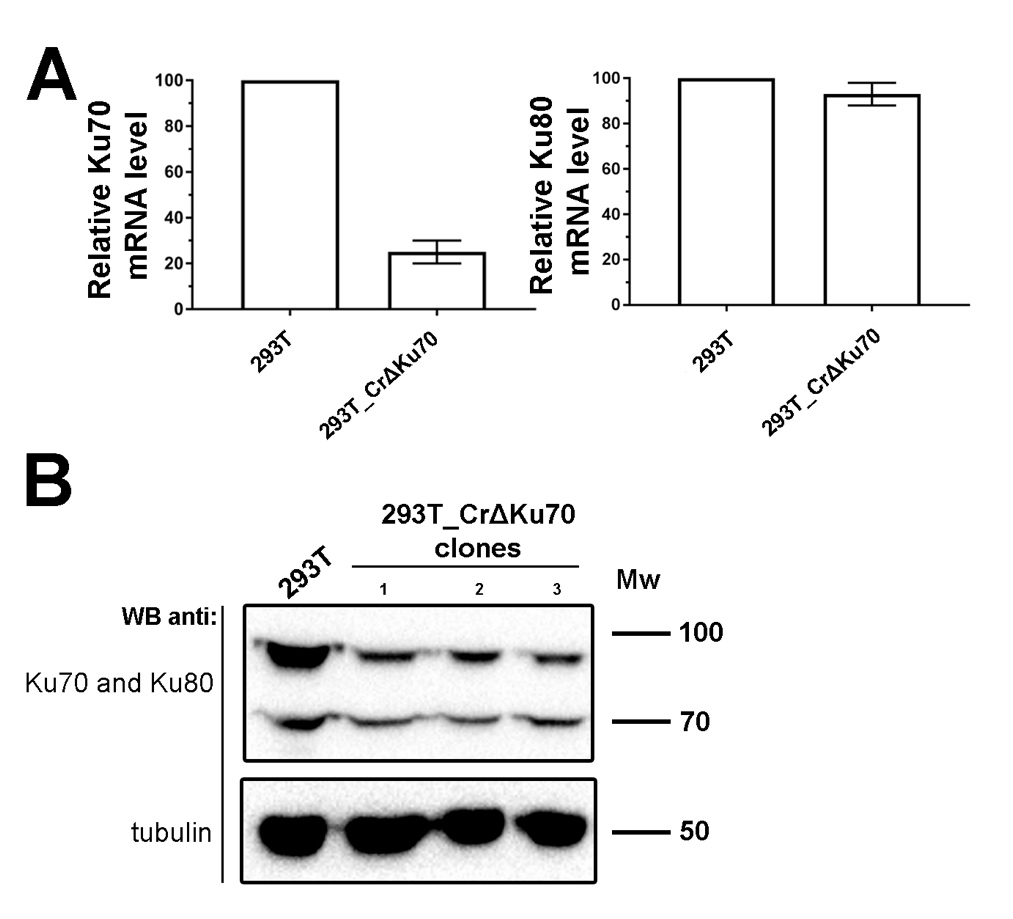
**

**Figure S3. Analysis of protein and mRNA levels in cells treated by CRISPR/Cas9 system.**

**(A)** A qPCR analysis of Ku70 (left panel) and Ku80 (right panel) mRNA level in 293T-Ku70+/- cells as compared to parental 293T cells. Means and SDs (n=3) are plotted. (**B)** Western blot analysis of 293T-Ku70+/- clones, tubulin is a loading control. The weight marker migration positions are presented to the right of the WB panels.


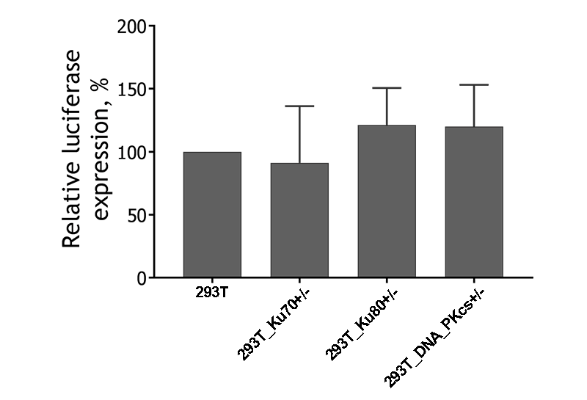


**Figure S4. Relative luciferase expression of Firefly luciferase from pGL3_CMV vector under the control of CMV promoter in 293T Ku70+/-, 293T-Ku80+/- and 293TCr∆DNA-PKcs+/- knocked down cell lines as compared to the expression level in parental 293T cells.**

Cells were transfected by pGL3_CMV expression vector together with a Renilla luciferase pRL_hPGK vector and 48h postransfection luciferase expression was analyzed. Signal from Firefly luciferase was normalized to the signal from Renilla luciferase, expressed from a cotransfected pRL_hPGK vector. Means and SDs (n=6) are plotted.


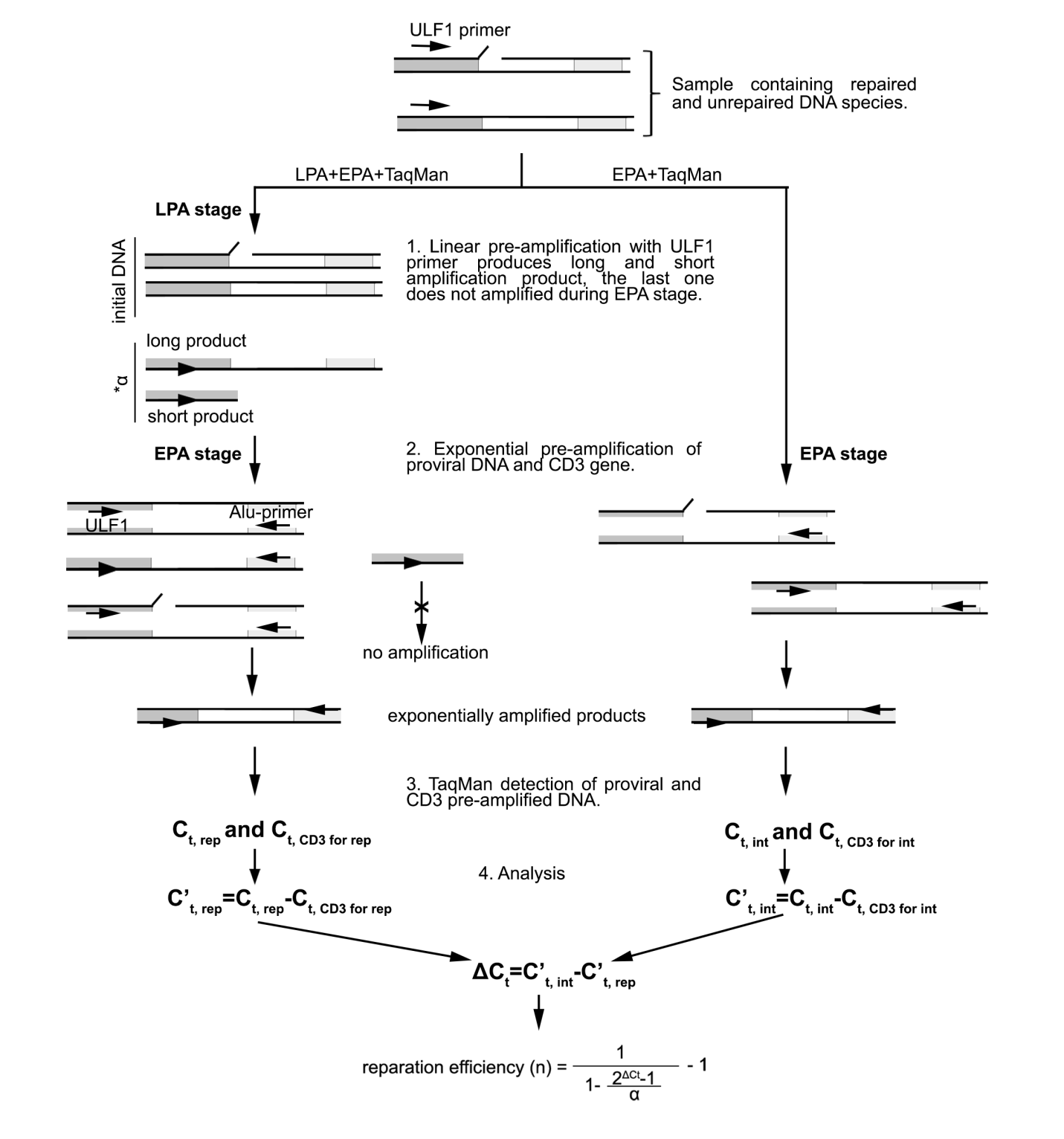


**Figure S5. General scheme of the PCR-based assays used to measure proviral DNA repair efficiency:** I-U – integrated unrepaired proviral DNA, I-R – integrated repaired proviral DNA, α – the number of cycles in linear pre-amplification stage, LPA – linear preamplification, EPA – exponential pre-amplification**.**

**
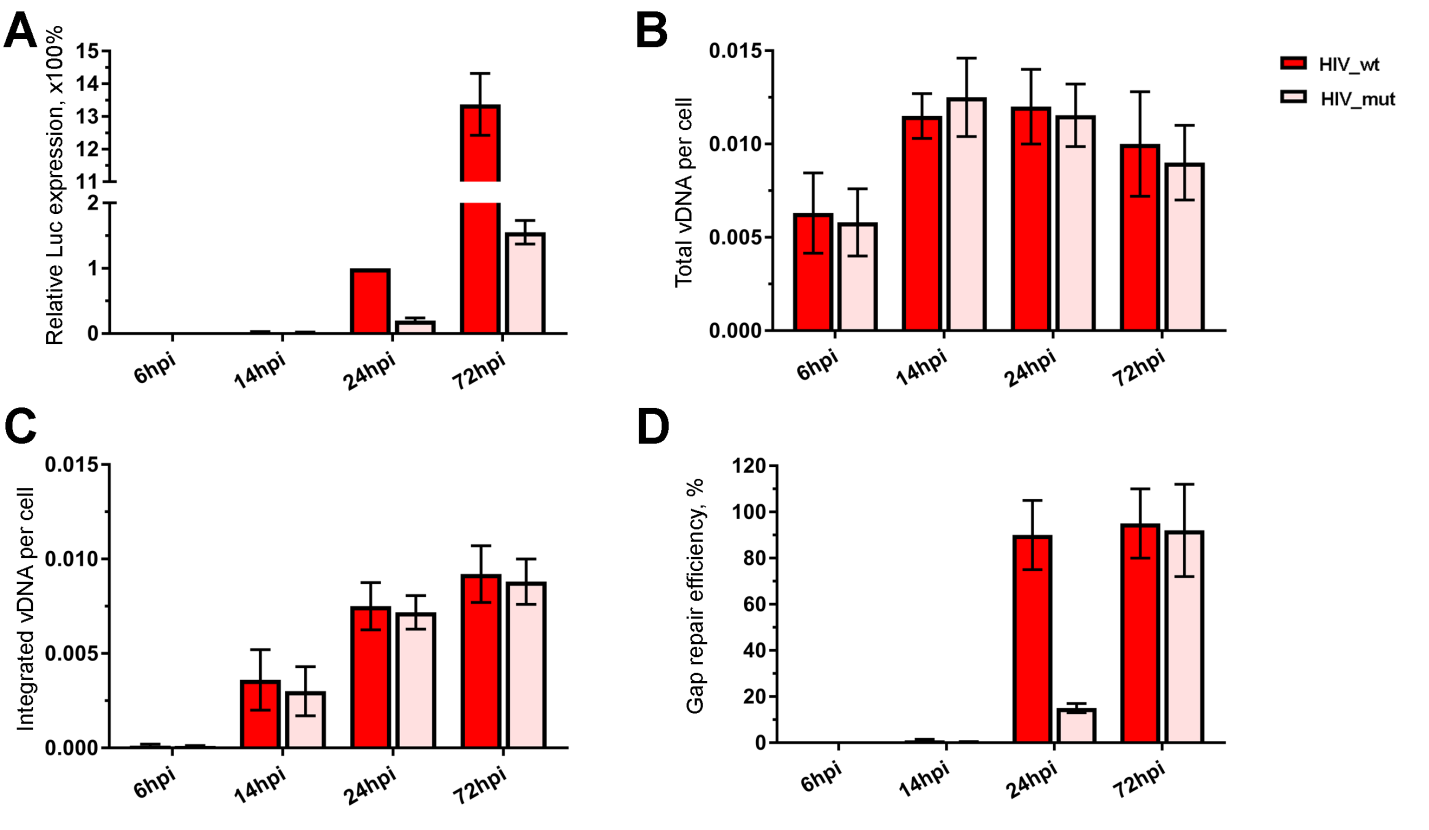
**

**Figure S6. Kinetic study of Luc expression (A), total (B), integrated viral DNA (C) and gap repair efficiency 6, 14, 24 and 72 hours post infection in HEK 293T cells.**

**
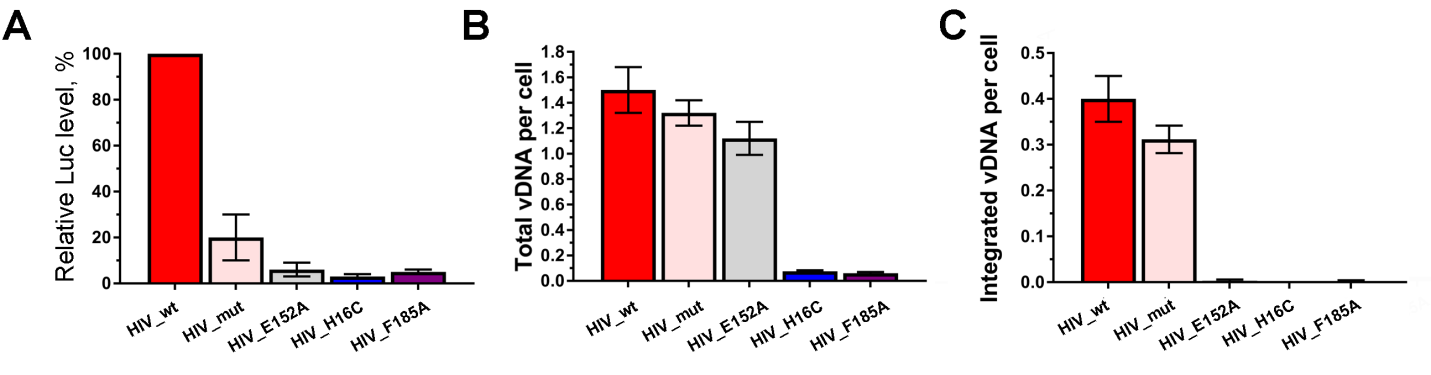
**

**Figure S7. Distinct effects of the class 1 and class 2 IN mutants on the early replicative stages of HIV-1 life cycle. (A)** Relative luciferase expression in 293T cells transduced by HIV_wt, HIV_mut, HIV_H16C, HIV_E152A or HIV_F185A pseudoviruses (MOI ~ 1.5) 24 h.p.i. **(B-C)** Levels of total viral DNA (**B**) and integrated viral DNA (**C**) in transduced 293T cells 24 h.p.i.

**
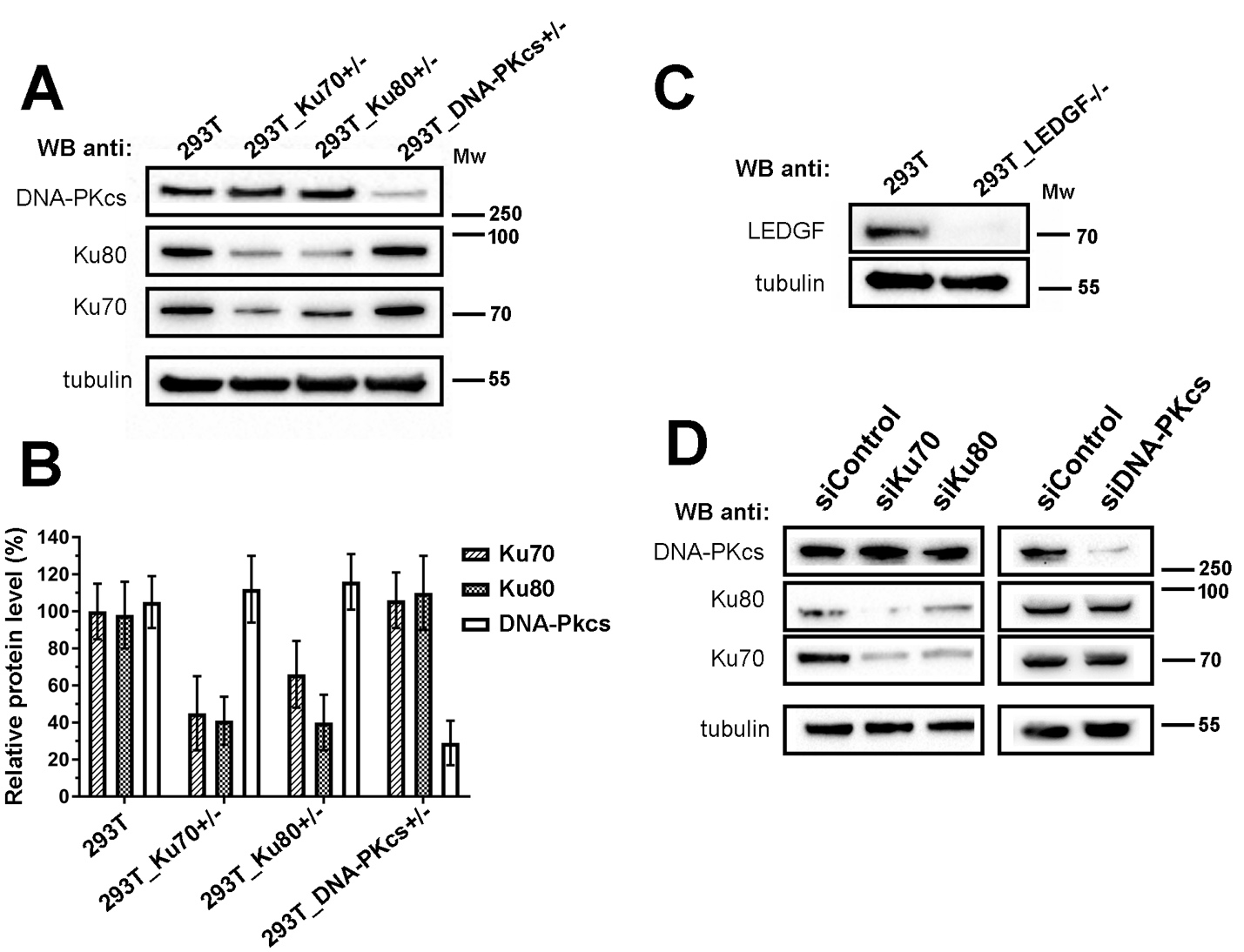
**

**Figure S8. Western blot analysis of the efficacy of proteins’ knockdown.**

**(A)** Representation of knockdown levels of Ku70, Ku80 and DNA-PKcs in respective CRISPR/Cas generated stable knockdown cell lines with tubulin as loading control. Representative image of three replicates is shown. (**B)** Quantification of the knockdown efficiencies displayed on A. **(C)** Representation of the knockdown of LEDGF/p75 in 293T-LEDGF-/- cell line with tubulin as loading control. (**D)** The efficacy of siRNA-mediated knockdown in 293T cells upon transfection of 50 nM of indicated siRNA. Cells were lysed and analyzed for target protein level 72 h after transfection. Tubulin is loading control. The weight marker mobility levels are presented to the right of the WB panels.

1. ^†^ The authors contributed equally to this work. [↑](#footnote-ref-1)
